# Activity and diversity of sulfate- and methane-based pathways of anaerobic chitin and N-acetylglucosamine degradation in marine sediments

**DOI:** 10.1128/aem.01280-26

**Published:** 2026-09-28

**Authors:** Sujung Lim, Ranjani Murali, Daan R. Speth, Rachel E. Szabo, Otto X. Cordero, Victoria J. Orphan

**Affiliations:** 1 Division of Geological and Planetary Sciences, Caltech 224033, Pasadena, California, USA; 2 Division of Biology and Biological Engineering, Caltech 124492, Pasadena, California, USA; 3 Department of Civil and Environmental Engineering, Massachusetts Institute of Technology 122707, Cambridge, Massachusetts, USA; University of Delaware, Lewes, Delaware, USA

**Keywords:** functional redundancy, community structure, methanogenesis, sulfate reduction

## Abstract

Sulfate reduction is estimated to account for half of the organic carbon respiration in anoxic, organic-rich ocean sediments. Sulfate-reducing microorganisms (SRMs) typically oxidize simple carbon compounds, such as primary alcohols, or small fatty acids. However, much of the organic input to the seafloor is complex, driving interactions between terminal respirers, such as SRMs and methanogens, and primary degraders that initiate the breakdown of complex organic carbon. To explore how these interactions shape sediment microbiomes, we conducted multi-month microcosm experiments using serially diluted sediments in deep 96-well plates. Sediments from a former deep-sea whalefall site were amended with either the insoluble polymer chitin or its soluble monomer N-acetylglucosamine. The high replication in these experiments allowed us to evaluate the effects of complex versus labile carbon on degradation activity, microbial diversity, community structure, and functional redundancy over time. Geochemical analysis, combined with 16S rRNA gene sequencing and metagenomics, revealed that chitin addition preserved higher microbial diversity and increased predicted interactions among microorganisms. Despite anaerobic chitin degradation producing N-acetylglucosamine, microbial communities enriched by these substrates showed low nestedness over the 7-month experiment. Complex carbon inputs fostered unique microbial assemblages and functional interactions, including the emergence of diverse methanogenic lineages at medium to high dilutions—less apparent in monomer treatments. In microoxic sediments, methanogens and other rare biosphere members co-exist with SRM and dynamically respond to complex carbon inputs. Our results highlight the contribution of carbon complexity and recalcitrance in stimulating metabolically diverse community members in sediments, driving the assembly of functional microbial networks.

**IMPORTANCE:**

Deep-sea sediments cover over 60% of the planet’s surface and harbor diverse microbial communities that are important contributors to the carbon and nitrogen budget of the ocean. Despite their importance, the mechanisms by which these communities maintain diversity are poorly understood. In this work, we discuss the contribution of complex carbon to the community structure of marine sediment microbial consortia through the establishment of highly replicated anaerobic incubations provided with either the complex cosmopolitan carbon source chitin or its monomer N-acetylglucosamine.

## INTRODUCTION

An estimated half of the organic matter degraded in marine sediments occurs through microbial sulfate reduction, making it the dominant respiratory process in global ocean sediments with fluxes of up to 11 Tmol sulfate reduced per year (1, 2). Microbial ecological surveys indicate that sulfate-reducing microorganisms (SRMs) dominate organic-rich sediments with as much as 85% abundance (3–5). The conventional understanding is that organic matter breakdown in anaerobic systems is coupled to a downward cascade through redox gradients as available oxidants are depleted in thermodynamic order (6). In anoxic ocean sediments, sulfate serves as the dominant terminal electron acceptor, and once it is depleted, methanogenesis becomes the primary respiratory process. However, organic matter present in sediments is often complex, polymeric, or aromatic, making it inaccessible for degradation by most SRMs or methanogens. These low-energy respirers must therefore depend on upstream processes, such as the production of exoenzymes by fermenting heterotrophs, which generate the simple fatty acids and alcohols necessary for their growth.

The aminopolysaccharide chitin (C_8_H_15_NO_6_)_n_ is an important constituent of complex particulate organic matter in marine environments. As one of the most abundant biopolymers in the ocean, it is a major contributor to global carbon and nitrogen cycles with an estimated annual production of over 10^13^ kg (7). The carapaces of copepods, crabs, and shrimp are commonly recognized as the major donor sources of chitin in the ocean, but its production encompasses all trophic levels as it is also synthesized by phototrophic diatoms (8, 9). Chitin has been found to be a major component of marine snow, with much of it mineralized aerobically during transit to the seafloor through the oxic water column (10, 11). As such, most environmental microcosm studies have examined the impact of chitin on aerobic communities (12, 13). Less is known about anaerobic degradation of chitin in marine sediment; in deep-sea productivity hotspots like whalefalls, crustaceans and other macrofauna with chitinous exoskeletons tend to congregate (14), resulting in localized deposition of chitin to the seafloor. Anaerobic chitin degradation studies in the laboratory have identified the following major steps. Primary chitin degraders, likely fermenters in anaerobic environments, produce exoenzymes allowing the initial cleavage of the chitin polymer to oligomeric forms (15). Chitin cleavage is followed by degradation of oligomers, including the monomer N-acetylglucosamine (GlcNAc), by primary and secondary chitin degraders. In anaerobic environments, secondary degraders produce organic acids and primary alcohols, which are available to the terminal respirers, in this case, either SRMs or methanogens (16, 17).

Marine sediments represent one of the most taxonomically diverse ecosystems, after terrestrial soils, providing many potential interactions within their microbial communities (18–20). These interactions appear to be promoted more by functional groups than taxonomic membership (21–24). This suggests that different species within a functional guild could readily substitute for each other within an ecosystem (“functional redundancy”), making it less likely that communities degrading chitin and GlcNAc are composed of a fixed set of core taxa. Sequencing-based surveys indicate a high level of predicted functional redundancy within terminal respirer groups such as SRMs and methanogens in marine sediments (4, 25, 26). While functional redundancy is thought to contribute to the maintenance of community structure in aerobic microcosms (27), its role in anaerobic deep-sea microbial communities is still unclear. Prior work suggests that a diffuse correlation network that contains multiple layers of nodes (members) and edges (interactions) is descriptive of the relationship between community interactions and the overall community response (28, 29).

Moreover, rare biosphere members—defined as less than 0.1% abundance—can have an outsized impact, providing key metabolic functions and allowing communities to remain resistant to perturbations while being maintained stably in their local environment (30–33). Prior work has established the importance of numerically rare taxa to biogeochemical cycling, especially with regard to SRM (34–36). Therefore, considering the apparent functional redundancy of multiple rare taxa of SRM or other terminal respirers may have important implications on overall ecosystem function and, more broadly, environmental impact.

Here, we tested the impact of organic matter complexity on marine sediment microbial communities over time, using microcosm incubations established using serially diluted deep-sea sediment from a former whalefall site. The design of our 7-month experiment enabled us to examine the fundamental roles of community structure and functional redundancy in metabolic guilds during the breakdown of the complex polymer chitin and its constituent monomer GlcNAc. Over the course of this experiment, we observed the impact of the dilution of rare community members on community-wide metabolic activity. In response to chitin amendment, we also observed a proliferation of key respirers such as methanogens and SRMs that were taxonomically similar to the original responders to the newly established whalefall nearly two decades prior. To reflect the low-temperature, low-energy regime on the seafloor, this experiment was carried out under psychrophilic and anaerobic conditions. Sub-sampling incubations over a time course enabled tracking the trajectory of metabolic potential and geochemical changes over the degradation of differentially complex carbon substrates. These microcosms were highly replicated, allowing us to disentangle stochastic effects caused by sediment heterogeneity from an actual signal of community structure.

## MATERIALS AND METHODS

Summarized materials and methods are included below. Detailed materials and methods are included in the supplemental text.

### Sample collection, incubation conditions, and media design

The sediment core was collected in December 2018 on Monterey Bay Aquarium Research Institute (MBARI) research cruise WF12-18 on the R/V *Western Flyer* with ROV *Doc Ricketts* at GPS coordinates 36.769873 N, 122.0829 W, and 1,018 m depth. After sectioning depth horizons from the sediment core, the 6–9 cm horizon was used as an inoculum. Tenfold serial dilutions of a 1:1 sediment slurry in 1.75 mL total volume were anaerobically prepared using a sulfate-free artificial seawater medium in 2 mL deep-well 96-well plates (VWR part number 89237-526). Four serial dilutions and up to 12 biological replicates were analyzed (Table 1; Fig. S1). Dilutions were done to simplify the input community and to assess the level of community reduction associated with the loss of carbon degradation and sulfate-reduction activity. This medium was supplemented with 5 mM sodium sulfate and, depending on treatment, colloidal chitin (5.7 mg/mL), N-acetylglucosamine (5 mM), or no electron donor. The medium recipe and preparation protocol for colloidal chitin are described in the supplemental text. The sulfate concentration chosen was below that of the initial site (∼28 mM) to prevent excessive sulfide accumulation within the incubations. After anaerobic incubation (ambient pressure) at 10°C for 1, 3, and 7 months, 0.3 mL from each well was subsampled for metagenome sequencing, 16S rRNA gene amplicon sequencing, and geochemistry by filtering through a 0.2-micron filter plate (Pall product number 8019). The filtrate was saved at −20°C for ion chromatography, and the filtrand was saved at −80°C for DNA extraction.

**TABLE 1:** Tabulated sample counts for geochemistry, 16S rRNA gene amplicon sequencing, and metagenomic sequencing^*a*^

Dil., months	Chitin			GlcNAc		
	*n*GC	*n*16S	*n*MG	*n*GC	*n*16S	*n*MG
Dil. 1 5 × 10^−5^, 1 mo	12	12	1	12	12	1
Dil. 1 5 × 10^−5^, 3 mos	12	11	1	12	9	1
Dil. 1 5 × 10^−5^, 7 mos	12	11	1	12	11	1
Dil. 2 5 × 10^−6^, 1 mo	12	12	1	12	11	1
Dil. 2 5 × 10^−6^, 3 mos	12	10	1	9	8	1
Dil. 2 5 × 10^−6^, 7 mos	12	11	1	12	11	1
Dil. 3 5 × 10^−7^, 1 mo	12	12	1	10	12	1
Dil. 3 5 × 10^−7^, 3 mos	12	11	1	11	5	1
Dil. 3 5 × 10^−7^, 7 mos	12	11	1	12	11	1
Dil. 4 5 × 10^−8^, 1 mo	4	12	1	12	12	1
Dil. 4 5 × 10^−8^, 3 mos	4	11	1	4	6	1
Dil. 4 5 × 10^−8^, 7 mos	4	11	1	4	12	1

^*a*^ GC, geochemistry; 16S, 16S rNRA gene amplicon sequencing; MG, metagenomic sequencing. Detailed by-plate sample information is provided in Fig. S1.

### Geochemistry: quantification of major ions and metabolites

Samples for ion chromatography were stored at −20°C until analysis, when they were diluted 1:50 and run on Dionex ICS-2000 or Thermo Integrion HPIC as previously described (37). These instruments are housed in the Water and Environment Laboratory (Caltech).

### DNA extraction and sequencing

Samples were extracted using the Beckman DNAdvance kit (Beckman product number A48705) using an adapted protocol (adapted from reference 38). Extracted DNA was quantified with PicoGreen (Invitrogen product number P7589), according to the manufacturer’s protocol. Samples were submitted for 16S rRNA gene sequencing of the V4 hypervariable region with Illumina adapters appended to the 5′ end of each primer set as described previously (37, 39). Primers used are described in the supplemental text. One replicate set from the DNA samples submitted for 16S rRNA gene sequencing was additionally submitted for metagenome sequencing to the MIT core sequencing facility (Cambridge, MA, USA) on a HiSeq 2500 (Illumina) after library preparation using the Nextera XT library preparation kit and quality control with a TapeStation 4200 (Agilent) using the HSDNA D1000 screen tape, both according to the manufacturer’s protocol. Library output per sample is included in the supplemental text (Table S4).

### 16S rRNA amplicon sequence analysis

Demultiplexed 16S rRNA amplicon reads were put through the QIIME2 pipeline with denoised ASV abundances generated using DADA2 (40, 41). Taxonomic classification was done using the SILVA SSU database release 138 (42, 43). Samples with fewer than 20 reads were removed from the analysis. A total of 880 ASVs were classified. Decontamination and abundance visualization of 16S data were done using the R (version 4.0.3) decontam and phyloseq packages (44, 45). Community structure, indicator species, and correlation analyses were done using the vegan, indicspecies, and fastspar packages (46–48). Visualization of networks was done using the igraph R package (49) and Cytoscape (50) with MCODE clustering (51). As a first pass for these clusters, possible sulfate-reducing and methanogenic taxa were putatively defined as members of the phylum *Desulfobacterota* or orders *Methanosarcinales*, *Methanofastidiosales* (formerly the WSA2 group [52]), and *Methanomicrobiales*, respectively (Data S2).

### Metagenomic analysis of metabolic marker genes

Metabolic marker genes for sulfate reduction (*dsrA*) and methanogenesis (*mcrA*) were extracted from metagenomic sequence reads using a previously described protocol (53) and manually curated databases derived from GTDB release 202 (Data S4 and S5). This approach was used due to the few high-quality bins derived from the metagenomic data (Table S5). A hidden Markov model (54) was prepared with HMMbuild (55) from taxonomically diverse DsrA and McrA amino acid sequences present in the IMG database (56) using BLASTP (57) followed by filtering with UCLUST (58). Metagenome reads were searched against the constructed database using DIAMOND (59). These reads were then searched against an outgroup consisting of the NCBI-nr protein reference database, and the BLAST score ratio (BSR) was calculated between the two searches; a BSR threshold of 0.75 was applied. Reference phylogenetic trees were built using a MUSCLE alignment (60) of the database sequences corresponding to mined metagenome sequences matching at least 95% identity with IQTREE (61), bootstrapped 100 times. Visualization of trees was done with iTOL (62).

## RESULTS

To examine the microbial community potential for the anaerobic degradation of the insoluble polymer chitin and its monomer GlcNAc, we established replicated anoxic microcosm experiments using serially diluted sediment inoculum from a previously described whalefall site in the Monterey Submarine Canyon, CA, USA at 1,018 m water depth (“Whale 1018” [63]). This study focused on a subset of dilutions and replicates from the larger experiment; all 12 replicates from 5 × 10^−5^ to 5 × 10^−7^ and four replicates from 5 × 10^−8^ (henceforth described as dilution 1 to dilution 4 in order of ascending dilution) were analyzed (Table 1; Fig. S1). We assessed the effect of treatment, incubation time, and dilution on microbial community structure and function by (i) measuring respiration of the provided substrate, (ii) 16S rRNA gene sequence-based analysis of the change in community membership and structure, and (iii) metabolic gene mining (53) of metagenomic sequences to assess the functional redundancy of SRM and methanogenic archaea.

### Geochemical proxies for microbial GlcNAc and chitin degradation

#### Metabolite production and sulfate drawdown highlight temporal and substrate-dependent dynamics in the carbon breakdown process

Chitin is an insoluble polymer, making it difficult to measure the top-down process of chitin degradation directly. We instead followed the accumulation of ammonium and acetate, the previously reported breakdown products of chitin and monomer GlcNAc, as a proxy for their degradation (11, 15). According to the following predicted stoichiometry of three moles acetate, one mole of ammonium, and eight reducing equivalents, GlcNAc oxidation is assumed to be coupled with equimolar amounts of sulfate reduction as the terminal respiratory process in these incubations. This results in the theoretical production of three moles acetate per mole of ammonium and a loss of one mole of sulfate per overall community reaction (hypothesized total reaction, equation 1).


(1) $$C_{8}H_{15}NO_{6}+2H_{2}O+SO_{4}^{2-}\to {3CH_{3}COO}^{-}+NH_{4}^{+}+3H^{+}+2HCO_{3}^{-}+HS^{-}$$

Using this idealized equation, we tracked the progression of chitin or GlcNAc oxidation over the course of 7 months; the 3:1 acetate:ammonium ratio is expected to change over time as secondary processes (e.g., acetate oxidation) occur. Indeed, we observed accumulation of these products in our incubations at a greater concentration than was seen in the source inoculum (Fig. S2).

**Fig 1:**
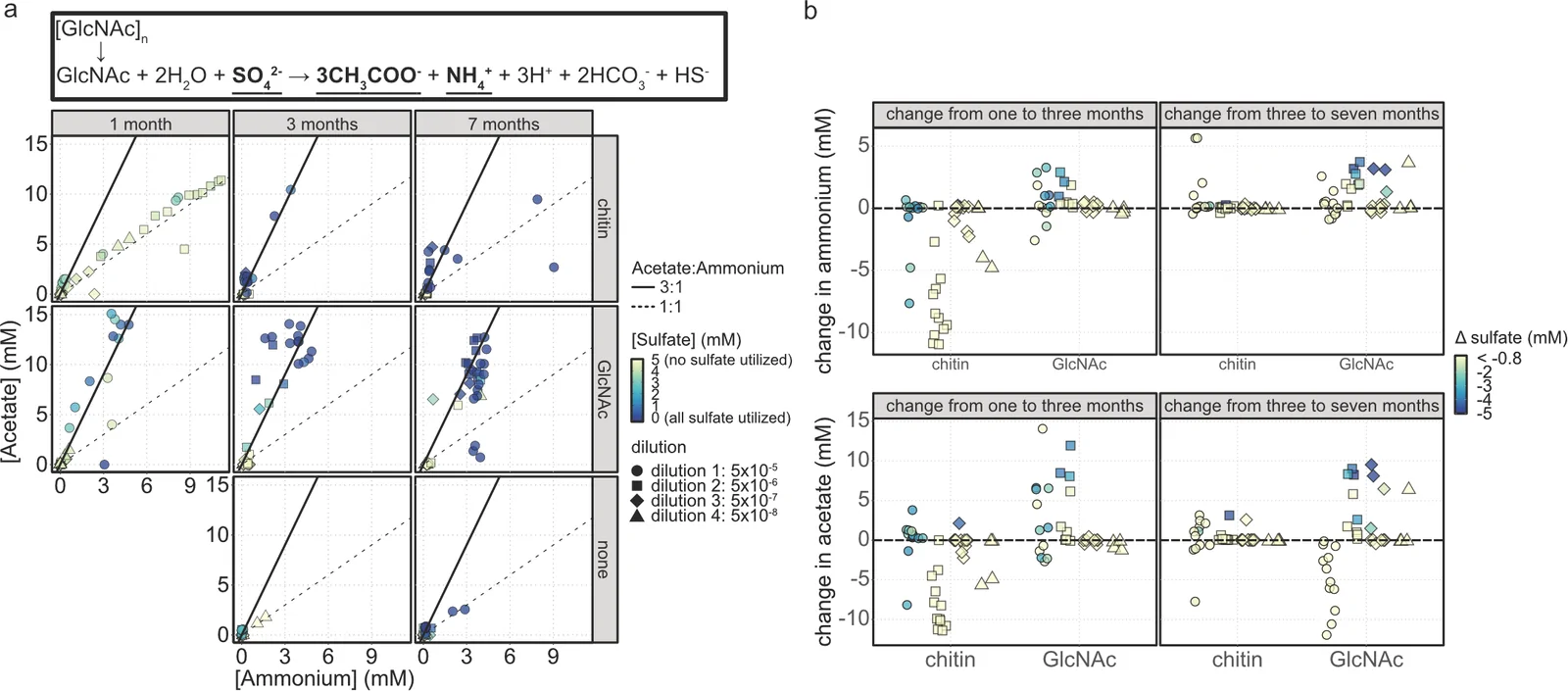
Patterns in metabolite production (ammonium and acetate) in 96-well microcosm experiments. (**a**) The bulk ratio of metabolic end products of a subset of dilutions (represented by point shape) and replicates demonstrates a temporal shift in the stoichiometric balance between ammonium and acetate. Catabolic processes additionally result in the drawdown of sulfate (color gradient) and acetate concentration. The solid 3:1 line denotes the ratio of acetate to ammonium if all of the carbon substrate is fermented or oxidized to acetate according to the stoichiometry of the hypothetical reaction listed above. Early chitin-degrading incubations exhibit an equimolar amount of excreted ammonium and acetate (dashed line). The initial metabolite concentration in the media was 0 mM for acetate and ammonium, and 5 mM for sulfate. (**b**) Difference plots of acetate and ammonium concentration illustrating a change in metabolite production/consumption between the first two (1–3 months) and last two (3–7 months) timepoints. Different dilution treatments are represented by shape. The horizontal line represents no change between timepoints, and shading represents differential sulfate concentration between the timepoints.

Ammonium utilization in these incubations is expected to be largely tied to anabolism, and drawdown occurs at a lower rate than catabolism of the other GlcNAc metabolic product, acetate. For this reason, the carbon degradation activity of the GlcNAc- and chitin-amended samples can be proxied by ammonium accumulation. We chose an activity threshold based on accumulation of the hypothesized metabolites acetate or ammonium above 0.5 mM or the utilization of sulfate below a measured concentration of 4.2 mM using the inflection points of metabolite concentration in an empirical cumulative density function plot (Fig. S3). In GlcNAc treatments, 60% of samples in dilutions 1–4 (69/115) met this activity threshold; these had a mean acetate:ammonium ratio that increased over the first two timepoints (1 month: 2.52:1, SD 1.6, *n* = 37; 3 months: 3.63:1, SD 2.15, *n* = 21) (Fig. 1a, middle row). The average ratio then decreased after 7 months (2.52:1, SD 1.65, *n* = 29), likely due to acetate utilization in conjunction with further ammonium accumulation.

Notably, a small number of GlcNAc-amended replicates (*n* = 3) showed active carbon metabolism that was uncoupled from a stoichiometric amount of sulfate drawdown by the final timepoint (Fig. 1a, middle row, right). Although sulfate was largely exhausted in most active microcosms by 3 months, acetate continued to decrease until 7 months (Fig. 1b), suggesting a potential for acetate-based syntrophy or direct consumption via acetoclastic methanogenesis.

Chitin-amended incubations contained fewer metabolically active samples, with 50% of samples meeting our activity threshold. These active incubations were heavily weighted to the two lowest dilutions analyzed (5 × 10^−5^ and 5 × 10^−6^) and additionally exhibited a strong shift in the ratio of metabolic products over time (Fig. 1a, top row). At the earliest timepoint, active samples that produced high amounts of acetate (>1 mM) had acetate:ammonium ratios clustering near 1:1 (average acetate:ammonium 1.05:1, SD 0.31, *n* = 19), far below the predicted acetate:ammonium ratio of 3:1. This indicated either enhanced acetate consumption over the first month or an alternative pathway for chitin degradation (Fig. 1a, top row, left). The ammonium production in these samples over the first month appeared decoupled from sulfate reduction, with little to no sulfate drawdown; by the 7-month timepoint, 14 out of 40 incubations exhibited >0.5 mM sulfate utilization. This time lag in sulfate-reducing activity may be explained by the limited accessibility and slower degradation of chitin as the insoluble polymeric electron donor. This suggests the potential involvement of additional processes beyond simple cleavage to the GlcNAc monomer and subsequent secondary oxidation. Acetate drawdown by other community members or acetoclastic methanogens could explain the deviation from the predicted 3:1 stoichiometry. Another mechanism could include the production of nonionic metabolites such as ethanol (proposed overall metabolic reaction, equation 2) or other small fatty acids in lieu of acetate. We did not test for ethanol, but fatty acid metabolites such as propionate or butyrate were not detected in the microcosms.


(2) $$[GlcNAc{]}_{n}\to GlcNAc+4H_{2}O\to CH_{3}COO^{-}+NH_{4}^{+}+2CH_{3}CH_{2}OH+2H^{+}+2HCO_{3}^{-}$$

Incubations that were not supplied with an external carbon source (the no carbon control) showed some evidence of metabolic activity, presumably from endogenous organic carbon degraded to acetate and ammonium. Production of these substrates was observed out to 3 months and at the highest sediment dilution (dilution 4) (Fig. 1a, bottom row), where little carryover of sediment from the initial inoculum is expected. The samples using endogenous carbon rather than supplied GlcNAc or chitin did not produce acetate or ammonium at the 3:1 predicted stoichiometry for GlcNAc degradation and had lower levels than those amended with chitin and GlcNAc, with a maximum of 2.56 mM acetate and 2.88 mM ammonium produced in one incubation replicate. The degradation of this endogenous carbon was coupled to sulfate drawdown at the final timepoint, with the two incubation wells that accumulated above-average ammonium additionally having completely consumed sulfate.

#### Sediment dilution has a pronounced effect on the metabolic response in GlcNAc- and chitin-amended incubations

We observed notable shifts in activity related to sediment dilution while tracking the progression of each GlcNAc treatment replicate over time from dilutions 1 through 4. The lowest dilution (dilution 1; 5 × 10^−5^) showed strong acetate drawdown, whereas higher dilutions accumulated up to 10 mM of acetate between the 3- and 7-month timepoints. Ammonium concentration trends were more variable between replicates, but on average, ammonium levels increased over the last two timepoints (Fig. 1b, top; Fig. S4), indicating continued substrate degradation. In some replicates, the increased accumulation of metabolites at high dilution coincided with the absence of sulfate drawdown (Fig. 1b, right), suggesting either a loss of active sulfate reducers or other key community members at higher sediment dilutions. In chitin-amended incubations, SRM in the high dilution microcosm samples appeared to have only a modest amount of activity based on the low level of sulfate utilization. This contrasted with the initial period of high acetate and ammonium production, followed by a subsequent drawdown. This rise-and-fall pattern was observed in all dilutions except dilution 1 during the first two timepoints (1–3 months). Both metabolites then accumulated between the last two timepoints (3–7 months), indicating a shift in community behavior after the third month (Fig. 1b).

### The stabilizing effect of complex carbon on community composition

#### The relationship between active carbon utilization and community composition over time

**Fig 2:**
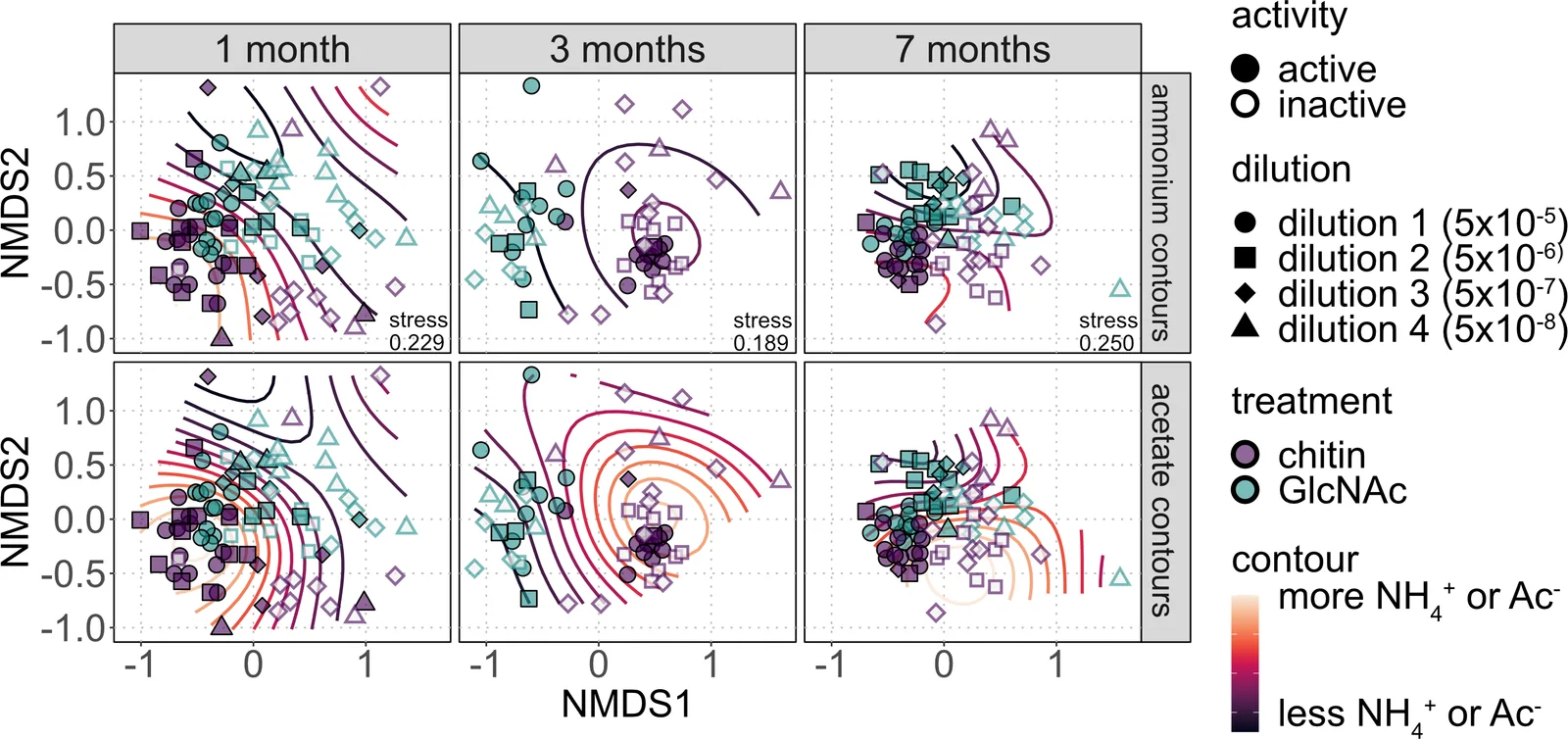
Community dissimilarity using nonmetric multidimensional dissimilarity analysis (NMDS). Only samples with geochemistry data are included in this analysis, and metabolite abundance is contoured on the plots (*P* < 0.005). The accumulation of both acetate and ammonium contributes to the dissimilarity between samples and varies at each timepoint. Meanwhile, per-treatment community dissimilarity converges over time. Contour lines follow ammonium (top) and acetate (bottom) concentrations, with yellow corresponding to greater metabolite concentration. Active samples are indicated by filled symbols, with activity thresholded to at least 0.5 mM accumulated ammonium or acetate, or at most 4.2 mM sulfate.

**Fig 3:**
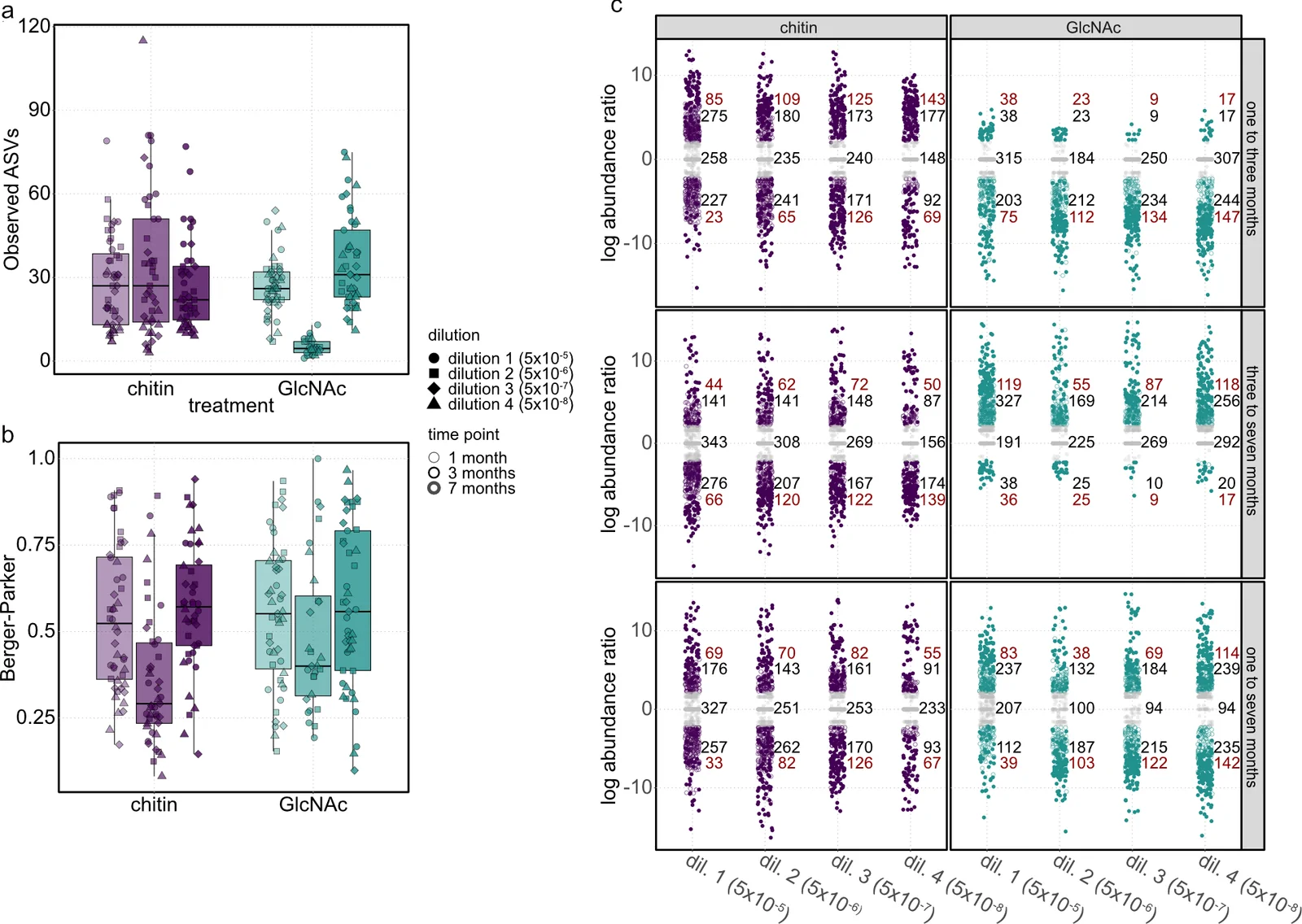
Substrate complexity has a stabilizing effect on community structure. (**a**) Box plots demonstrate higher richness over the first two timepoints in chitin-amended treatments relative to GlcNAc. Chitin-amended treatments at low dilution maintained their richness over time (point and box opacity) in contrast with GlcNAc-amended treatments, which exhibited a diversity crash at the second timepoint. Per-dilution mean diversity metrics (observed ASVs and Berger-Parker dominance) are included in Table S2. (**b**) Berger-Parker dominance metrics do not demonstrate strong trends in evenness between treatments except for the 3-month timepoints in both treatments, which are weighted toward lower evenness. (**c**) Community difference plots following genus gain and loss measured by log_2_(*x* + 1) absolute abundance change between pairs of timepoints. Filled points correspond to “rare” genera that were below 0.1% relative abundance; point color corresponds to treatment for genera that exhibited at least a twofold abundance change. Counts of rare genera with at least twofold abundance changes are included in red, and total counts of each group are in black.

The variation in metabolic activity across dilutions, combined with the complexity of the amended carbon source, allowed us to examine the relationships between microbial activity and community composition. Genus-level binning of the 16S rRNA gene sequence data simplified downstream analysis while maintaining a high level of sampling completeness (Fig. S3). Nonmetric multidimensional scaling (NMDS) analysis showed that GlcNAc- and chitin-degrading communities became increasingly dissimilar over time before eventually converging at the 7-month mark. Comparison with geochemical data revealed that acetate and ammonium accumulation contributed to the dissimilarity in community composition between treatments, particularly at the early timepoints (Fig. 3; Table S1). When we assessed the dissimilarity of all treatments, the trend observed was consistent with the patterns seen in the metabolic outputs. Specifically, the 1-month chitin incubations at all dilutions exhibited greater dissimilarity from all other timepoints and incubations, consistent with the unusual acetate:ammonium ratios (Fig. S6). While these early timepoint chitin microcosms clustered together and were distant from other treatments, by 7 months the actively metabolizing microbial community had converged (Fig. 2). Communities in incubations which lacked a carbon amendment did not exhibit this convergence (Fig. S6).

This difference was also observed in ANOSIM analysis of replicates from the active microcosms with statistically significant dissimilarity between chitin and GlcNAc communities increasing from 0.1875 at 1 month to 0.611 at 3 months (Table S1). The difference at 7 months (−0.035) was insignificant. Follow-up similarity percentage (SIMPER) analysis on significant ANOSIM analyses demonstrated that the top 30% taxa with significant (*P* < 0.05) contributions to dissimilarity between chitin and GlcNAc treatments at the 1-month timepoint were *Psychromonas*, *Desulfuromonadales* family Sva1033, and *Pontiella* (formerly Kiritimatiellales family R76-B128 [64]); at the 3-month timepoint, these were *Desulfoconvexum*, *Carboxylicivirga*, an unassigned member of the class *Bacteroidia*, and *Marinifilum* (Data S1). The major contributors to dissimilarity at both timepoints were largely related to known psychrophiles, consistent with the deep-sea source inoculum and incubation temperature. These taxa were on average more abundant in GlcNAc treatments in the last two timepoints. The above taxa, except for Sva1033 and *Desulfoconvexum,* are also groups known to contain heterotrophic bacteria that have the potential to degrade not only mono- and disaccharides but, surprisingly, also polysaccharides of varying complexity. The metabolic potential and ecological roles for these genera were inferred from cultivated species and environmental surveys (64–69). We could identify specific “indicator taxa” (70) associated with the active incubations at nearly all timepoints which are further discussed in the supplemental text.

#### Effect of carbon complexity and dilution on community structure and association networks

Chitin amendments supported higher levels of microbial diversity, especially at the lower dilutions, compared with GlcNAc amendments in the first 3 months. By the final, 7-month timepoint, however, the species richness and evenness converged between chitin and GlcNAc treatments (Fig. 3a and b; Table S2). GlcNAc amendments exhibited a precipitous decrease in richness and unique taxa between the first two timepoints followed by a rebound between the last two timepoints (Fig. 3c). In comparison, while the input sediment inoculum was richer than chitin-amended incubations, they were at the same order of magnitude (Fig. S7). The chitin-amended incubations additionally exhibited a modest change in richness over the course of the experiment. This change masked a dynamic gain and loss of taxa in the incubations over time. There was gain and loss of genera at roughly equal magnitude during both the first two (1–3 months) and last two (3–7 months) timepoints in the chitin-amended replicates, and, corresponding with NMDS analysis, the communities at the first and final timepoints were dissimilar.

**Fig 4:**
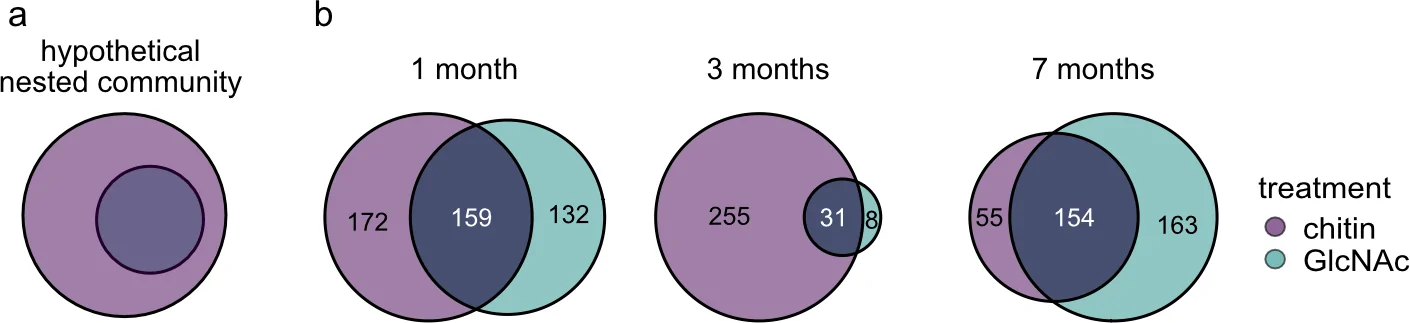
Monomer-amended communities are not compositionally nested within polymer-amended communities. (**a**) In a hypothetical nested community, those carrying out downstream metabolic processes, such as GlcNAc degradation, would be a subset of the chitin-degrading community. (**b**) Euler diagrams show that GlcNAc-amended community members present across all replicates and dilutions at each timepoint are not nested within chitin-amended communities. Numbers represent the number of genera identified within each treatment.

Many of the genera gained over the course of the experiment originated from a rare state with less than 0.1% relative abundance (Fig. 3b; Fig. S9). The increased diversity in early chitin amendments suggested that the polymer-degrading communities may represent a superset of the monomer-degrading communities (e.g., Fig. 4a). However, when we assessed the pool of shared genera within each treatment to make a conservative estimate of the “core microbiome,” we did not observe compositional nesting between GlcNAc- and chitin-amended microcosm communities (Fig. 4). This was contrary to our expectation that the community composition in these incubations would mirror the nestedness of the GlcNAc and chitin degradation processes.

Given the role of sulfate reduction as the major respiratory process in these incubations, we predicted there would be a correlation between sulfate reduction activity and community structure. However, no correlation was observed between sulfate utilization and overall diversity in any treatment (Table S3). Furthermore, despite our expectation that sulfate-reducing taxa would be associated with sulfate drawdown, DNA-based evidence of putative sulfate-reducing taxa still occurred in high-dilution microcosms (dilutions 3 and 4), where sulfate utilization was minimal or absent. For example, while sulfate respiration in the chitin treatment was primarily observed in the lowest dilution treatments (5 × 10^−5^; Fig. 1b), putative sulfate-reducing 16S rRNA lineages persisted even at the highest dilution (dilution 4, 5 × 10^−8^) (Fig. S10). A similar pattern was also observed in metagenomic data with the detection of dissimilatory sulfite reductases (*dsrA*) at the highest dilution.

**Fig 5:**
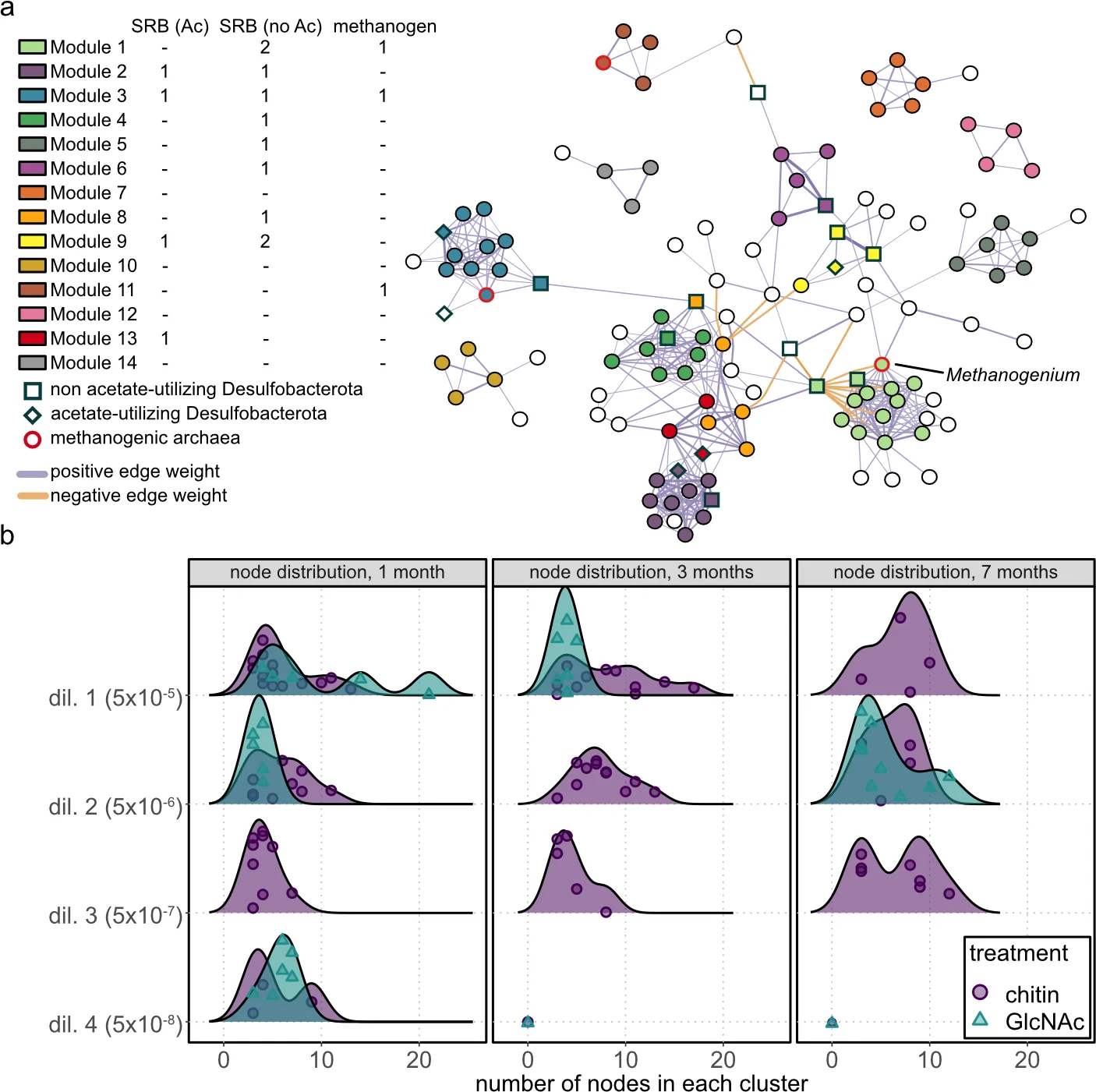
Statistically significant (*P* < 0.05) co-occurrence networks generated with fastspar on microbial assemblages from geochemically active incubations separated by dilution, treatment, and time. (**a**) Example network from the chitin-amended incubation at 1 month of incubation and dilution 1. Modules generated from MCODE clusters (Data S3) are shown with colored points, and the number of inferred SRB (acetate-utilizing and non-acetate-utilizing) and methanogens per module are listed. Rectangle and diamond node shapes represent inferred acetate utilization in genera within the *Desulfobacterota*. The hydrogenotrophic methanogen genus *Methanogenium* is labeled; all other methanogens in the example network shown are inferred methylotrophic or acetoclastic. (**b**) Frequency distribution plot (legend, inset) describing the number of nodes per cluster in co-occurrence networks generated with fastspar on active populations separated by dilution, treatment, and timepoint. The highest dilutions at the last two timepoints had no clusters.

To determine whether microcosms with the highest activity contained common interaction patterns between distinct community members, we generated co-occurrence networks using the active incubations for each carbon amendment, dilution, and timepoint. All statistically significant networks (*P* < 0.005, threshold 0.5) contained putative sulfate-reducing taxa consistent with the sulfate reduction activity observed. Sulfate-reducing taxa were also present in the network for the early timepoint of the active chitin incubations preceding observed sulfate drawdown. In all but two dilution/timepoint pairs of this set, chitin incubations each contained a higher number of discrete clusters than GlcNAc treatments (Fig. 5b), consistent with the greater overall diversity observed in these incubations. Furthermore, occurrence of the hydrogenotrophic methanogen *Methanogenium* in correlation networks was often associated with putative incomplete acetate-oxidizing SRM (e.g., *Desulfoconvexum*) in active incubations (Data S3; full Cytoscape network data at https://doi.org/10.22002/4raz4-aa715). Other methanogenic genera such as methylotrophic *Methanococcoides* and *Methanosarcina* were also present in correlation networks for nearly all dilutions of chitin-treated incubations, suggesting the presence of viable methanogens *in situ* and potential interactions with other community members.

### The effect of carbon amendment on the functional redundancy of terminal respirers such as SRM and methanogens

#### High diversity of sulfate-reducing taxa supported polymer chitin amendments over the monomer GlcNAc

**Fig 6:**
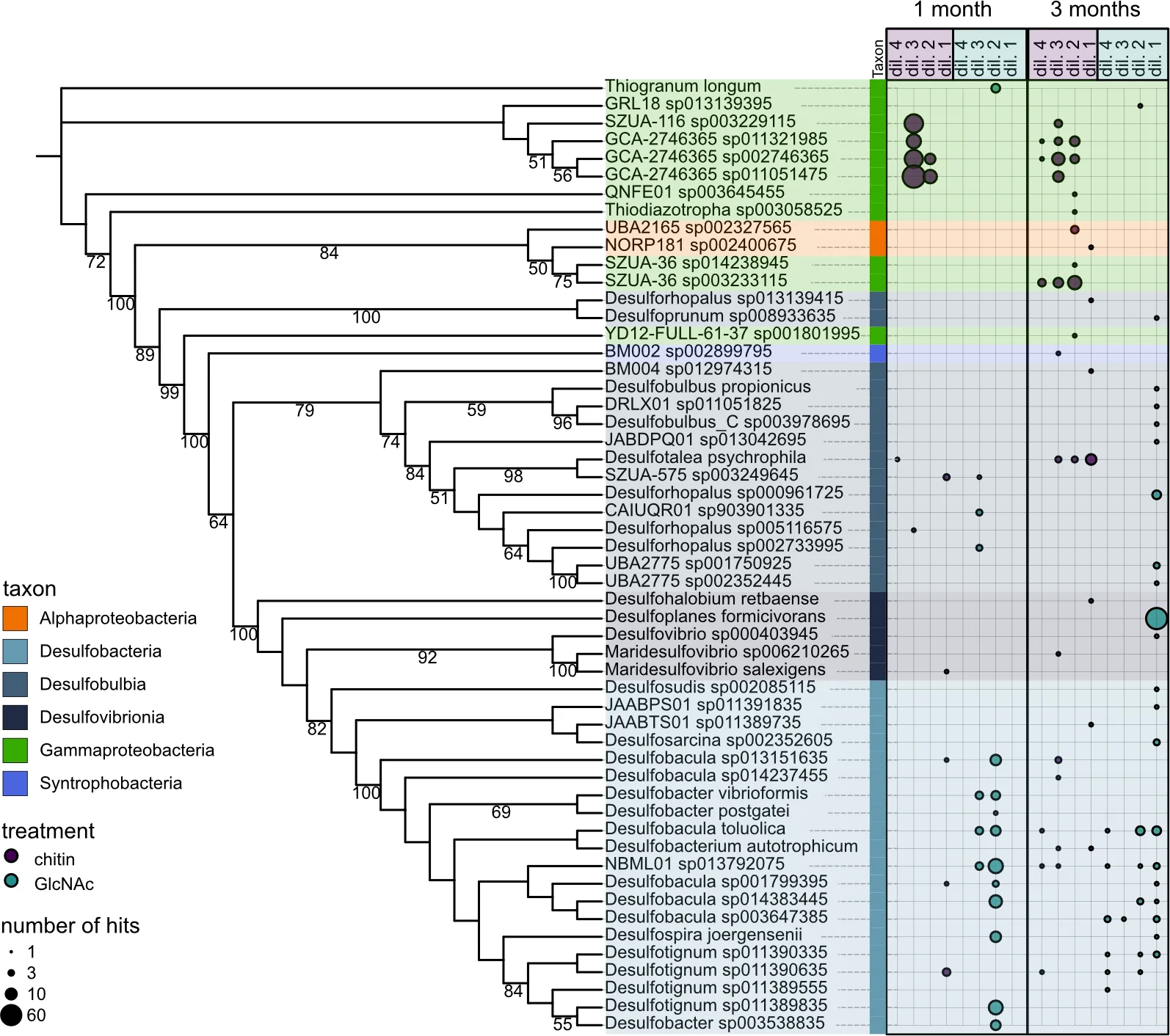
Phylogenetic tree showing the distribution of metagenome sequence hits mined from a GTDB DsrA amino acid database. GTDB DsrA sequences were aligned with MUSCLE, and the tree was generated with the IQ-TREE model LG+R6. Node labels correspond to bootstrap support values greater than 50%. Tip labels correspond to species from GTDB, while tips and horizontal shading are colored by taxon, with higher taxonomic resolution in the *Desulfobacterota*. The faceted plot to the right contains a summary of the metagenomic sequence distribution per treatment (Point color) separated by dilution and time. Point size indicates the number of reads corresponding to at least a 95% identity match with the GTDB amino acid database.

The dynamism in community data recorded by 16S rRNA gene amplicon sequencing was also independently supported by diversity and abundance of key genes for sulfate reduction (dsrA) and methanogenesis (mcrA) in metagenomic data. At the 1- and 3-month timepoints, we sequenced the metagenome of one of the 12 replicate samples from each serial dilution, representing four metagenomes per treatment and per timepoint and sixteen metagenomes total (eight per treatment). We coassembled these metagenomes to preliminarily assess the diversity of genes involved in the chitin and GlcNAc metabolic pathways. As expected, chitinase genes are found to be more common in chitin-amended treatments (Data S6). However, our metagenomes were found to have insufficient read depth, as illustrated by SingleM analysis (71), showing only 74 genera versus 134 genera extracted from 16S rRNA gene data on the same data set, and had few high-quality bins (Tables S4 and S5; Date S7). Therefore, we performed a read-based analysis on respiratory genes to support our 16S rRNA gene sequence data instead as we felt it was more illustrative of the diversity of our samples. The *dsrA* gene, a subunit of sulfite reductase, encodes the final step of sulfate reduction and is present in all putative sulfate reducers and some sulfur oxidizers with a high degree of homology (72). Identifying *dsrA* reads in the metagenome provided an assessment of within-guild diversity and possible functional redundancy across coexisting sulfate-reducing populations. We recovered *dsrA* from all treatments and dilutions; all four dilutions experienced a proliferation of phylogenetically distinct *dsrA* over the first two timepoints which was enhanced in chitin incubations (*n* = 65) vs in GlcNAc incubations (*n* = 55) (Fig. 6). There were more *dsrA* genes normalized by read number in chitin amendments than in GlcNAc amendments (Fig. S11), with three of the four chitin incubations exhibiting an increase in *dsrA* diversity, with an average 82% increase in unique *dsrA* genes, between the 1-month and 3-month incubation period. In contrast, *dsrA* diversity in GlcNAc incubations increased in only two of four incubations and averaged less than half the increase observed in the chitin incubations, at 38%. The overall trends seen in *dsrA* diversity with respect to treatment were consistent with other genes in the sulfate reduction pathway (*aprA, dsrB*; Fig. S12 to S14).

#### Methanogen proliferation in chitin treatments after sulfate depletion

**Fig 7:**
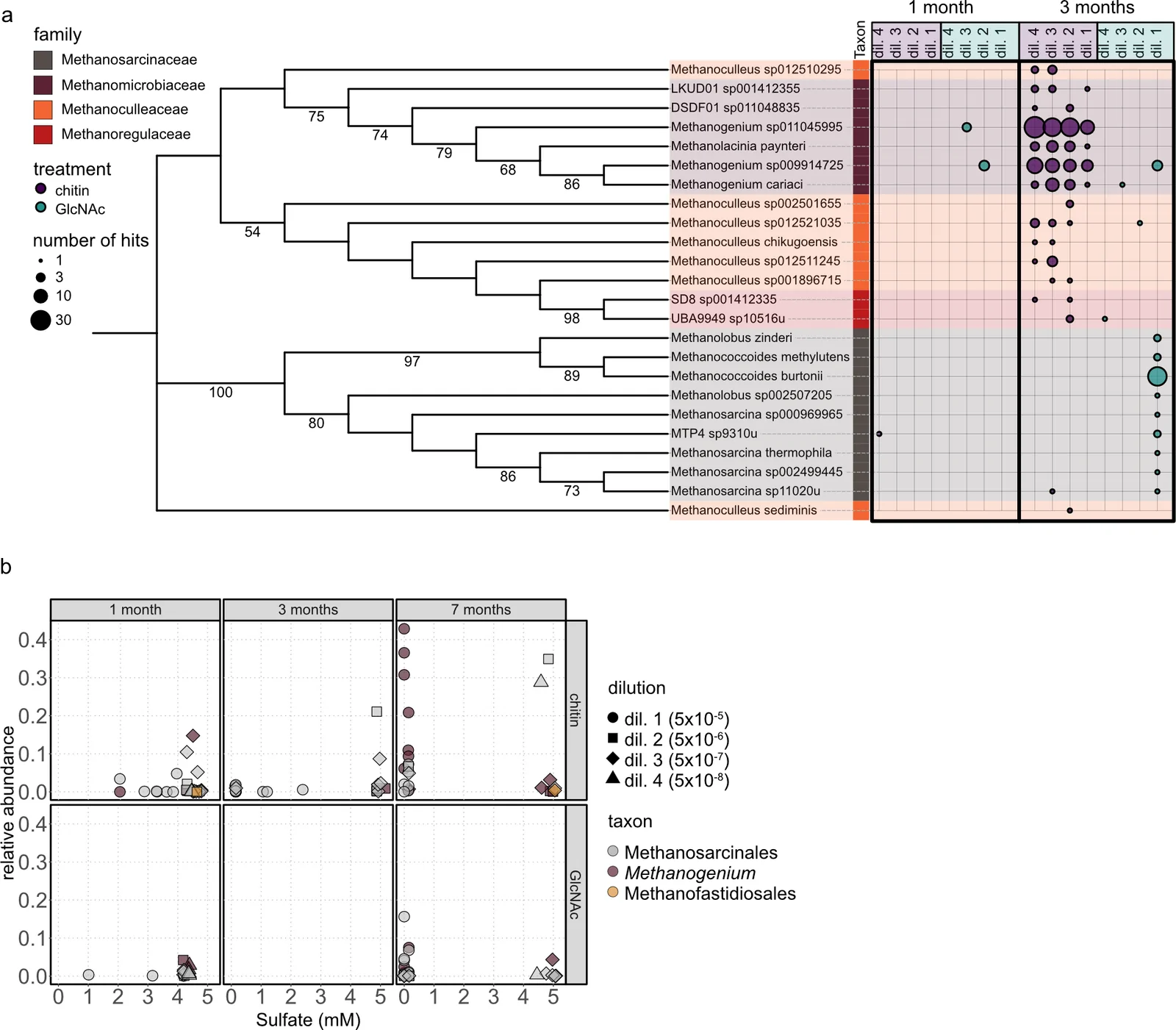
Molecular assessments of methanogen distribution. (**a**) Phylogenetic tree of sequence hits from a GTDB McrA amino acid database with dot plot showing the distribution of metagenome hits between incubation treatments. GTDB McrA sequences were aligned with MUSCLE, and the tree was generated with the IQ-TREE model LG+F+G4. Node labels correspond to bootstrap support values greater than 50%. Tip labels correspond to species from GTDB, while tips and horizontal shading are colored by family. Point color in the faceted plot to the right corresponds to treatment, and size indicates the number of reads corresponding to hits with at least 95% identity. Timepoints are distributed horizontally; the 7-month timepoint was not collected. (**b**) The relative abundance of putative methanogenic clades in 16S rRNA data for each incubation is plotted against sulfate concentration. Points are colored by methanogenic clade and shaped by timepoint. Significant negative correlations (*P* < 0.05) with sulfate concentration are with chitin at 7 months (Spearman’s *ρ_Methanogenium_* = −0.719, *ρ* all methanogenic clades = −0.686).

Differences in methanogenic clade abundance between treatments and timepoints were supported by functional gene data. We analyzed the presence of the methyl coenzyme reductase gene (*mcrA*), present in all methanogenic and methanotrophic archaea, from the metagenomic data. Consistent with the 16S rRNA gene amplicon data, the taxonomic assignment of *mcrA* varied by carbon treatment. *Methanogenium* was more abundant in the chitin treatments compared to GlcNAc, as observed in both the 16S amplicon and metagenomic sequence data. Interestingly, *Methanogenium mcrA* was detected earlier in the metagenomic data (at 3 months) than in the 16S rRNA amplicon data (at 7 months) (Fig. 7). Cultured *Methanogenium* species are known hydrogenotrophic methanogens and are not typically associated with noncompetitive substrate use (73, 74); thus, we predicted the occurrence of *Methanogenium* would coincide with timepoints after the exhaustion of sulfate. Indeed, at the final timepoint, *Methanogenium* 16S abundance was negatively correlated with sulfate concentration (*P* < 0.05, Spearman’s *ρ* = −0.719) when aggregating all dilutions and replicates in the chitin treatment. *Methanogenium* 16S abundance did not correlate with sulfate utilization in any other treatment or timepoint, consistent with the indicator taxon analysis (Fig. 7b). Although *Methanogenium* was not detected in the specific sediment depth horizon used as the inoculum for this experiment (Fig. S10), it was present at roughly 6% relative abundance in 16S rRNA gene surveys of a deeper horizon in the same sediment core (Fig. S15). Other methanogen lineages present at higher abundance in chitin treatments include members within the orders *Methanofastidiosales* and *Methanosarcinales* (Fig. 7b).

## DISCUSSION

The anaerobic breakdown of complex polymers, such as chitin, by microorganisms in deep-sea sediments remains largely unexplored. In this study, we employed a highly replicated and structured system to simultaneously track geochemical output and community dynamics over time, providing insights into the behavior of anaerobic marine sediment communities. By analyzing compositional and metabolic changes, we assessed the impact of carbon complexity on microbial community structure, the role of functional redundancy among metabolic guilds in maintaining community performance, and the relationship between community structure and activity. Despite the expected influence of stochastic processes on community heterogeneity (75), we observed a consistent association between the addition of complex carbon and the maintenance of community diversity (e.g., Fig. 3a, 5 and 6). This pattern may be driven by potential microbial interactions, such as syntrophy, along with physical factors, including the insolubility of the substrate and the spatial heterogeneity of the sediment inoculum (18, 76).

Anaerobic chitin degradation in freshwater systems has been shown to proceed through the hydrolysis of chitin to oligomers, such as chitobiose and GlcNAc, which are then fermented into small fatty acids or primary alcohols. Further oxidation steps can then occur coupled to the reduction of available electron acceptors, including sulfate (15, 77). While previous work using an inoculum from the same whalefall site has demonstrated stimulation of exoenzyme activity after chitin supplementation (76), we found in our experiments that substrate degradation and electron acceptor respiration do not necessarily occur simultaneously. For example, while GlcNAc incubations produced the predicted 3:1 stoichiometry between acetate and ammonium, chitin-amended microcosms showed significant accumulation of both acetate and ammonium, which was initially decoupled from sulfate reduction with an observed 1:1 acetate:ammonium ratio (Fig. 1a). In contrast, GlcNAc incubations exhibited sulfate-coupled substrate utilization (Fig. 1a). Prior work with freshwater sediment microcosms demonstrated extremely low acetate:ammonium ratios in response to chitin or GlcNAc amendment (<0.05 for chitin and <1 for GlcNAc [77]), in partial agreement with our data. While the low acetate:ammonium ratio observed in the chitin treatments was consistent with our work, the freshwater microcosms, regardless of substrate, exhibited much less acetate accumulation overall relative to ammonium.

The observed geochemical response by the microbial community, deviating from the 3:1 acetate-to-ammonium ratio in these deep-sea chitin microcosm incubations, suggests the anaerobic degradation process is more complex than simple hydrolysis to the monomer GlcNAc. This implies either substantial biological acetate consumption during the early phase of chitin incubation or the occurrence of fermentative processes which produce alternative metabolic products, such as primary alcohols, rather than acetate. While not common, the generation of primary alcohols from chitin has been previously described in an isolated member of the phylum *Fibrobacteres* (78). Members of the same candidate group (TG3) were observed in the chitin-amended incubations, suggesting that these alternative products could be part of a viable pathway for chitin degradation. The limited sulfate reduction observed was insufficient to account for the extent of acetate oxidation (over 10 mM, Fig. 1b) in a large subset of the chitin-amended incubations. Acetate could instead be consumed via pathways like acetoclastic methanogenesis or syntrophic acetate oxidation. At the low temperatures (10°C) used in our long-term incubations, acetoclastic methanogenesis is predicted to be favored over syntrophic acetate oxidation (79), and we see evidence of acetoclastic methanogen taxa in both 16S rRNA amplicon and metagenomic sequence data. Within some chitin-amended incubations, we observed a decrease in both sulfate and ammonium between the first and third months (Fig. 1b) that could potentially be explained through the process of sulfate-coupled ammonium oxidation (i.e., sulfammox) (80).

Since GlcNAc is a monomer of chitin, we initially hypothesized that the pathways and microbial communities involved in GlcNAc degradation would be a subset of those responsible for chitin degradation. However, our analysis revealed that while the communities became more similar over time (decrease in dissimilarity; Table S1), the community structure in the GlcNAc and chitin treatments was not nested by either composition or presumed function (e.g., Fig. 1 and 4). Previous studies have shown that the introduction of complex carbon (high molecular weight [HMW] polysaccharides) to environmental communities can promote “selfish” uptake by primary degraders, rather than competition among secondary degraders for degradation products, although this behavior is heterogeneously distributed (81, 82). While we do not know whether a similar strategy is occurring in our system with the insoluble polymer chitin, one possibility is that in the presence of complex carbon, primary degraders have an advantage over generalist microbes that typically utilize oligomers or monomers. In a mixed polysaccharide-degrading community, these generalist degraders may be outcompeted by primary degraders that preferentially consume complex carbon. Additionally, the extracellular hydrolysis of HMW polymers during primary degradation acts as a rate-limiting step that downstream metabolic reactions depend upon (83). Previously observed geochemical shifts, which suggested primary alcohol production in chitin-degrading incubations but not in GlcNAc-degrading incubations, may be attributed to alternative pathways associated with the more structurally complex substrate. Such pathway diversity may help explain the enhanced community complexity observed in the chitin amendment, as well as its divergence from the community enriched on the simpler GlcNAc monomer. This behavior is consistent with observations of shifts in community structure reported from other “complex” vs “simple” amendments in freshwater sediments and mixed cultures (chitin vs GlcNAc, and cellobiose vs glucose [75, 77, 84]).

In natural ecosystems, shifts in community structure over time can result from various ecological factors, such as dispersal, drift, or selection (85). However, in our experimental setup, each replicated microcosm functioned as a closed system, eliminating emigration and immigration over the 7-month experiment. In such closed systems, diversity may be sustained through spatial or metabolic partitioning (86, 87). In our chitin-amended incubations, we observed that both compositional and functional diversity were retained over time and with increasing dilution, although the variation increased in microcosms at higher levels of dilution (Fig. 3A). Essential members required for insoluble polymer degradation were maintained even at high inoculum dilutions (up to dilution 3) as assessed from the corresponding geochemical activity data, and community richness remained largely consistent despite several-fold dilution of the initial sediment inoculum. This pattern of diversity distribution across sediment particles aligns with trends observed in other spatially heterogeneous communities, such as those associated with sand particles (18). The ability of individual particles to retain a substantial portion of overall community diversity may act as a “seedbank,” enabling community resilience and success in highly dynamic and heterogeneous environments.

Although microbial community richness was enhanced and maintained across dilutions in chitin-amended microcosms, our experiments did not reveal a strong correlation between alpha diversity and the activity of the terminal respiratory process of sulfate reduction. This finding is consistent with previous studies that have reported an ambiguous relationship between alpha diversity and ecosystem function or activity (75, 88, 89). While individual core metabolic processes, such as substrate degradation and respiration (e.g., measured by carbon dioxide production), may be influenced by changes in community richness (88, 90, 91), other reports, particularly from freshwater microcosms amended with cellobiose or glucose, suggest that diversity is not directly linked to overall ecosystem performance. Instead, taxonomic diversity serves primarily as a descriptive metric for comparing treatments or conditions, rather than as a predictor of community-wide function (75, 89).

In our chitin-amended incubations, the stable alpha-diversity observed over the 7-month experiment was underlain by dynamic gains and losses of microbial genera over time (Fig. 3b), with turnover being more pronounced in chitin compared to GlcNAc incubations. This dynamic response was a common characteristic of complex carbon in these communities. In the absence of dispersal as a driver of diversity proliferation in these microcosms, the observed transitions between genera underscore the role of complex carbon in promoting active community turnover.

In addition to dynamic turnover of genera in chitin incubations, we also identified several indicator taxa which were only enriched in active microcosms amended with this complex carbon source (Fig. S8). This included members of the genus *Pontiella*, an organosulfur-degrading bacterial lineage (64), and methanogenic genera *Methanogenium* and *Methanococcoides* (73, 92). The potential production and utilization of sulfur compounds by *Pontiella* could promote further niche partitioning (e.g., through the production of sulfated polysaccharides [64]), contributing to the diverse community composition and interactions during anaerobic chitin degradation. Overall, our findings indicate that the community involved in anaerobic chitin respiration in these systems differs from the GlcNAc-degrading assemblage, with organosulfur-metabolizing taxa and distinct methanogen clades as distinguishing members. Beyond the key indicator taxa identified in chitin-amended microcosms, our observations also highlight the dynamic role of rare biosphere members, which responded uniquely to chitin treatments compared to the GlcNAc monomer and contributed to the stability and adaptability of the microbial community (Fig. 3b). Many of the terminal respirers (e.g., sulfate-reducing microorganisms and methanogens) that proliferated during the experiment, particularly those recovered from treatments with active carbon degradation, were either below detection or exceedingly rare in the initial inoculum (Fig. S10). Rare biosphere members are thought to enhance ecosystem adaptability by increasing resistance to environmental perturbations (35, 93–95), and their contribution to maintaining diversity in our long-term experiment supports this role (Fig. 3b). Although sulfate-reducing bacteria and methanogenic archaea can be abundant in marine sediments (4, 96), our source sediment collected from a deep-sea whalefall had not experienced a significant influx of organic matter for nearly two decades, when the whale was implanted (63, 96, 97). While active methanogenesis and sulfate reduction occurred over several years at the whalefall site, at the time of sampling no sulfide production was detected (Fig. S2) (96, 97). Despite the oxic sediment conditions during collection, it appears that the sediment assemblage retained a reservoir of viable methanogens and SRM that were taxonomically similar to those previously observed at this site (96, 97). This suggested a conserved “seedbank” response to complex carbon amendment, where the persistence of rare microbiome members, including obligate anaerobes, may contribute to community resilience and stability (94, 98).

Sulfate reduction ability, as the predominant anaerobic respiratory process in the oceans, was expected to be a major contributor to the community response in our experiments. Indeed, we observed a proliferation of functional diversity through taxonomically distinct *dsrA* genes in the chitin-amended incubations (Fig. 6; Fig. S11). The SRM containing these taxonomically distinct *dsrA* genes were also predicted to fill separate ecological niches, with some capable of completely oxidizing acetate and others unable to oxidize acetate. There appears to be a lack of correlation between acetate consumption and sulfate utilization between 1 and 3 months (Fig. 1b), suggesting that SRM here use substrates besides acetate, contrasting with prior reports from marine sediments where SRM primarily use small organic acids (99, 100). We additionally observed the effect of dilution on sulfate reduction presumably as abundant or highly active SRM are diluted out, with sulfate reduction persisting in GlcNAc treatments up to dilution 3 and only up to dilution 2 in chitin treatments (Fig. 1b). Despite the absence of sulfate drawdown, potential SRM as inferred by *dsrA* presence occurred even at the highest dilution samples (Fig. 6; Fig. S10 and S11). Whether these *dsrA*-containing lineages are incapable of sulfate reduction, engaged in an alternate metabolism, or below a critical threshold for activity remains to be determined. This may be due to the presence of inactive or metabolically flexible SRMs, organisms containing an incomplete sulfate reduction pathway, or potentially relic DNA, though the latter is unlikely due to the observed shift in *dsrA* diversity between dilutions (Fig. 6).

Alongside the dynamics observed among sulfate-reducing taxa, methanogenic taxa also emerged as a distinguishing feature between the chitin- and GlcNAc-amended microcosms, contributing uniquely to the community structure and metabolic processes observed over time (Fig. 5 and 7). Both SRM and some methanogens share common substrates such as acetate or hydrogen, and under environmental conditions, SRM are expected to outcompete acetoclastic or hydrogenotrophic methanogens due to their higher substrate affinity and greater energetic yield (74). Indeed, in our chitin-amended experiments, *Methanogenium*, an archaeal genus with hydrogenotrophic methanogens as the only known members, did not increase in 16S rRNA gene abundance until sulfate was exhausted (Fig. 7b), while methylotrophic methanogen taxa containing members known to use noncompetitive substrates such as methylamine (101) were present at high sulfate concentration (Fig. 7b), potentially coexisting with SRM. Chitin treatments additionally stimulated a member of the archaeal family *Methanofastidiosales*, a novel methylotrophic methanogen taxon capable of utilizing methylated thiols (52). The presence of *mcrA* genes phylogenetically associated with hydrogenotrophic genera in our metagenomes preceded the detection by 16S rRNA (Fig. 7a). Whether this was due to the slow proliferation of stimulated methanogens or the differential sensitivity of the two sequencing methods is unclear. Regardless, there was a clear signal of complex carbon stimulating diverse methanogens able to fill multiple ecological niches (Fig. 7). The monomer GlcNAc incubations additionally harbored low abundances of distinct methylotrophic taxa with little stimulation of hydrogenotrophs (Fig. 7a), in contrast to the chitin treatments.

While our results suggest that functionally diverse SRM and methanogens co-occur in these deep-sea sediments under both chitin and GlcNAc degradation conditions, it is unclear whether there are persistent ecological interactions that drive such redundancy. Using community correlation analysis (48), we were able to investigate potential microbial interactions within our polymer-amended microcosms, offering deeper insights into community dynamics. In our microcosm treatments, microbial communities were organized into co-occurring units (“modules”), each containing terminal respirers sulfate reducers or methanogens—along with other taxa, such as putative fermenters and general heterotrophs. These modules exhibited positive co-occurrence patterns, suggestive of as yet undefined interactions between members, such as trophic relationships, nutrient dependencies, and niche partitioning (28, 102, 103). Importantly, putative sulfate-reducing and methanogenic taxa were consistently found within these modules even at high dilution (up to dilution 3) within the active microcosms for both chitin and GlcNAc treatments, with the complex polymer treatment supporting a broader range of interactions within individual modules (Fig. 5). Modules within the chitin amendment incubation facilitated both functional diversity (through the proliferation and redundancy of metabolic genes—for example, genes involved in sulfate reduction) as well as interaction diversity (through the maintenance of multiple yet distinct co-occurrence modules). This modular structure was a common characteristic of the microbial communities involved in complex carbon degradation in our experiments and may be an intrinsic feature of anaerobic polymer degradation. Further research should aim to clarify whether the module patterns seen in functional group representation apply to communities utilizing other complex carbon sources and to uncover the mechanisms that support the formation and stability of these modules.

In this study, we used highly replicated and serially diluted microcosm experiments to examine the effect of carbon source complexity on the activity and structure of sulfate-reducing marine sediment microbial communities. We identified several key features of this anaerobic sediment community over the course of a 7-month incubation experiment, including the apparent retention of a diverse community associated with particles and the maintenance of functional complexity during the degradation of complex substrates, which contribute to biodiversity and functional redundancy in cold, anoxic deep-sea environments. Our findings reveal that complex carbon supports diverse terminal respirers such as sulfate reducers that organize into distinct groups with complex interactions between members. The persistence of diverse but rare marine methylotrophic and hydrogenotrophic methanogenic communities in oxic environments that are then capable of proliferating in response to organic matter inputs underscores their potential role in methane emission within near-seabed sediments that experience periodic pulses of complex carbon. The potential for similar processes across diverse environments—from nearshore ecosystems to the deep sea—highlights the dynamic role of functionally and taxonomically diverse sediment microbial communities, especially in climate processes (104, 105). Together, these insights contribute to our understanding of microbial community dynamics in anoxic sediments and underscore the importance of these communities in global carbon cycling.

## Data Availability

Raw sequence data are available in the Sequence Read Archive under accession number PRJNA1099895. Cytoscape network data are available at https://doi.org/10.22002/4raz4-aa715.

## SUPPLEMENTAL MATERIAL

Data S1 aem.01280-26-s0001.xlsx SIMPER analysis on significant ANOSIM analyses listing contribution of different genera to dissimilarity between chitin and GlcNAc treatments at each timepoint.

Data S2 aem.01280-26-s0002.xlsx Tabulation of the taxonomy data assigned to the "methanogen" or "SRB" functional groups.

Data S3 aem.01280-26-s0003.xlsx Tabulation of the data found in Fig. 5, including the total number of MCODE-generated clusters, the total number of clusters containing nodes with methanogenic taxa, and the total number of clusters containing nodes in the Desulfobacterota for each treatment, timepoint, and dilution.

Data S4 aem.01280-26-s0004.txt GTDB release 202 amino acid sequences of DsrA pulled using a constructed DsrA HMM.

Data S5 aem.01280-26-s0005.txt GTDB release 202 amino acid sequences of McrA pulled using a constructed McrA HMM.

Data S6 aem.01280-26-s0006.xlsx Effective gene diversity of genes involved in chitin and GlcNAc metabolism across samples.

Data S7 aem.01280-26-s0007.xlsx SingleM output containing relative abundance of taxonomy data present in each sample.

Supplemental material aem.01280-26-s0008.pdf Fig. S1 to S15, Tables S1 to S5, supplemental text and methods, and legends for Data S1 to S7.

ASM does not own the copyrights to Supplemental Material that may be linked to, or accessed through, an article. The authors have granted ASM a non-exclusive, world-wide license to publish the Supplemental Material files. Please contact the corresponding author directly for reuse.
