# Supplementary material for: Activity and diversity of sulfate- and methane-based pathways of anaerobic chitin and N-acetylglucosamine degradation in marine sediments
Source: Appl Environ Microbiol. 2026 Sep 28;92(10):e01280-26. doi: 10.1128/aem.01280-26 (PMC13689031; doi:10.1128/aem.01280-26)
Supplement: Supplementary file 1 [file aem.01280-26-s0008.pdf]

Supplemental material

Contents

Figure S1. Experimental diagram and sample processing scheme for experiment. 4

Figure S2. Porewater geochemistry from a paired sediment push core at the whale fall site used in this experiment. 5

Figure S3. Empirical cumulative density functions describing the distribution of metabolite concentration. 5

Figure S4. Per-well changes in ammonium and acetate between one and seven months. 6

Figure S5. Taxon accumulation plot for 16S sequencing data at the ASV or genus level. 7

Figure S6. NMDS plot with all samples containing geochemistry data plotted in the same pane including contours of metabolite concentration or dilution. 8

Figure S7. Plot of incubation and source inoculum Chao1 richness. 9

Figure S8. Differentially abundant genera associated with metabolic activity as determined by indicator taxon analysis. 10

Figure S9. Shifts in rare genus relative abundance over time, separated by general temporal trends. 10

Figure S10. Relative abundance of inferred methanogenic and sulfate-reducing taxa with 16S sequencing data. **12**

Figure S11. Distribution of mined sulfite reductase genes (*dsrA*) within each dilution for each treatment. **13**

Figure S12. Distribution of other sulfate reduction pathway genes *aprA* and *dsrB* between each dilution and treatment. **14**

Figure S13. Phylogenetic tree showing the distribution of metagenome sequence hits mined from a GTDB AprA amino acid database. **15**

Figure S14. Phylogenetic tree showing the distribution of metagenome sequence hits mined from a GTDB DsrB amino acid database. **16**

Figure S15. Relative abundance of *Methanogenium* within the inoculum sediment core. **17**

Table S1. Per-treatment community dissimilarity metrics (ANOSIM R statistic). **18**

Table S2. Means and standard deviations of per-dilution Chao1 richness. **20**

Table S3. Multiple linear regression analysis values showing the lack of correlation between sulfate concentration and diversity. **20**

Table S4. Illumina read output per sample. **21**

Table S5. Tabulated metagenome bins. **22**

Supplemental text. Indicator taxon analysis of active incubations. **23**

Supplemental methods **24**

Description of supplemental files **28**

References **29**

Supplemental Figures and Tables

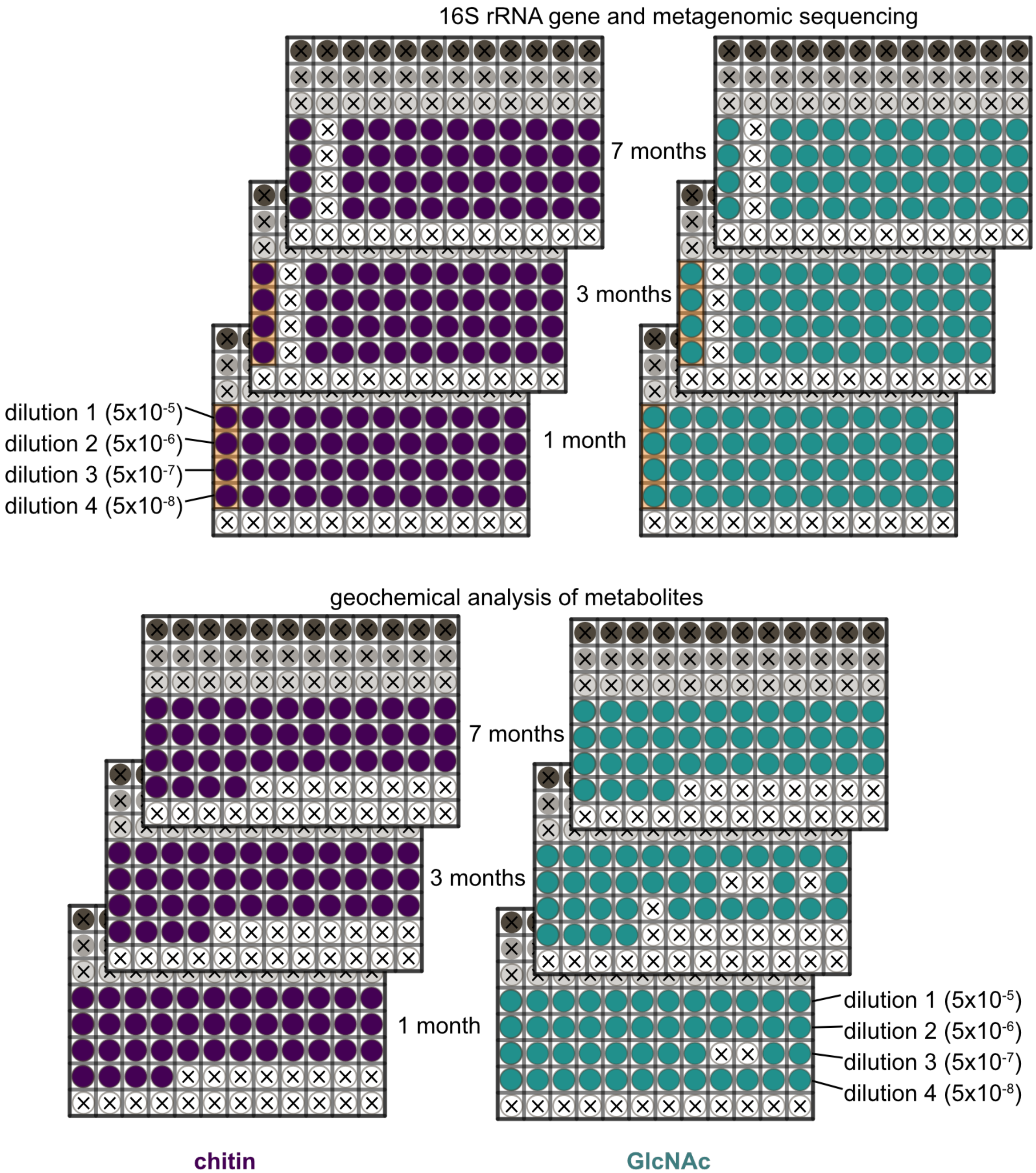

FIG S1 Experimental diagram and sample processing scheme for 16S rRNA gene amplicon sequencing (top, filled wells), metagenomic sequencing (top, orange highlights), and geochemical analysis of metabolites acetate, ammonium, and sulfate (bottom, filled wells). Wells marked with "x" were not analyzed in this study.

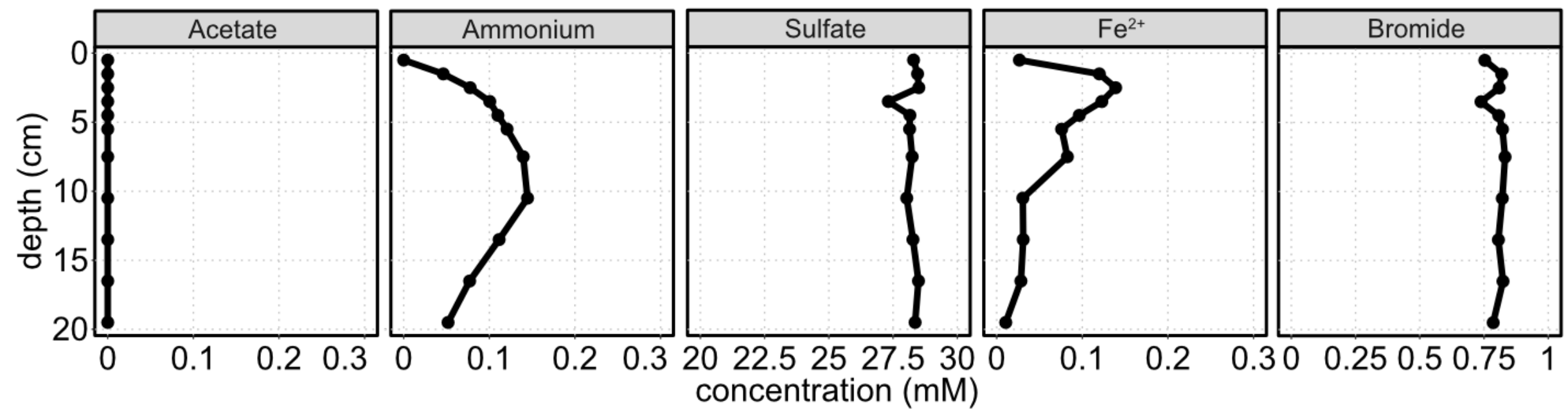

FIG S2 Porewater geochemistry from a paired sediment push core at the whale fall site used in this experiment.

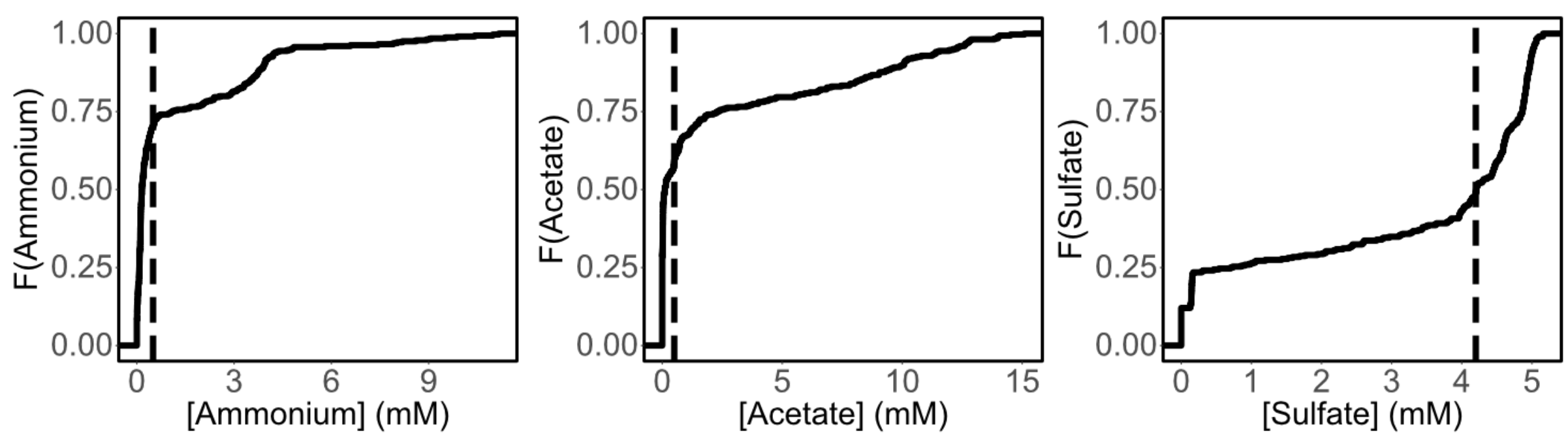

**Treatment**	**Time point**	**n** **$[NH_4^+]$ > 0.5 mM**	**n** **$[Ac^-]$ > 0.5 mM**	**n** **$[SO_4^{2-}]$<4.2 mM**	**n** **active**
None (n.m.)	1 month	n.m.	n.m.	n.m.	n.m.
None (n=40)	3 months	2	7	17	19
None (n=40)	7 months	3	13	22	22
GlcNAc (n=48)	1 month	13	15	33	23
GlcNAc (n=36)	3 months	20	19	18	21
GlcNAc (n=40)	7 months	29	28	26	29
Chitin (n=40)	1 month	20	25	15	29
Chitin (n=40)	3 months	4	12	13	14
Chitin (n=40)	7 months	5	14	14	15

FIG S3 Empirical cumulative density functions describing the distribution of metabolite concentration. Activity thresholds are shown with vertical lines: ammonium and acetate thresholds are set at 0.5 mM; and sulfate is thresholded at 4.2 mM. Table below lists incubation counts meeting each threshold. n.m., not measured.

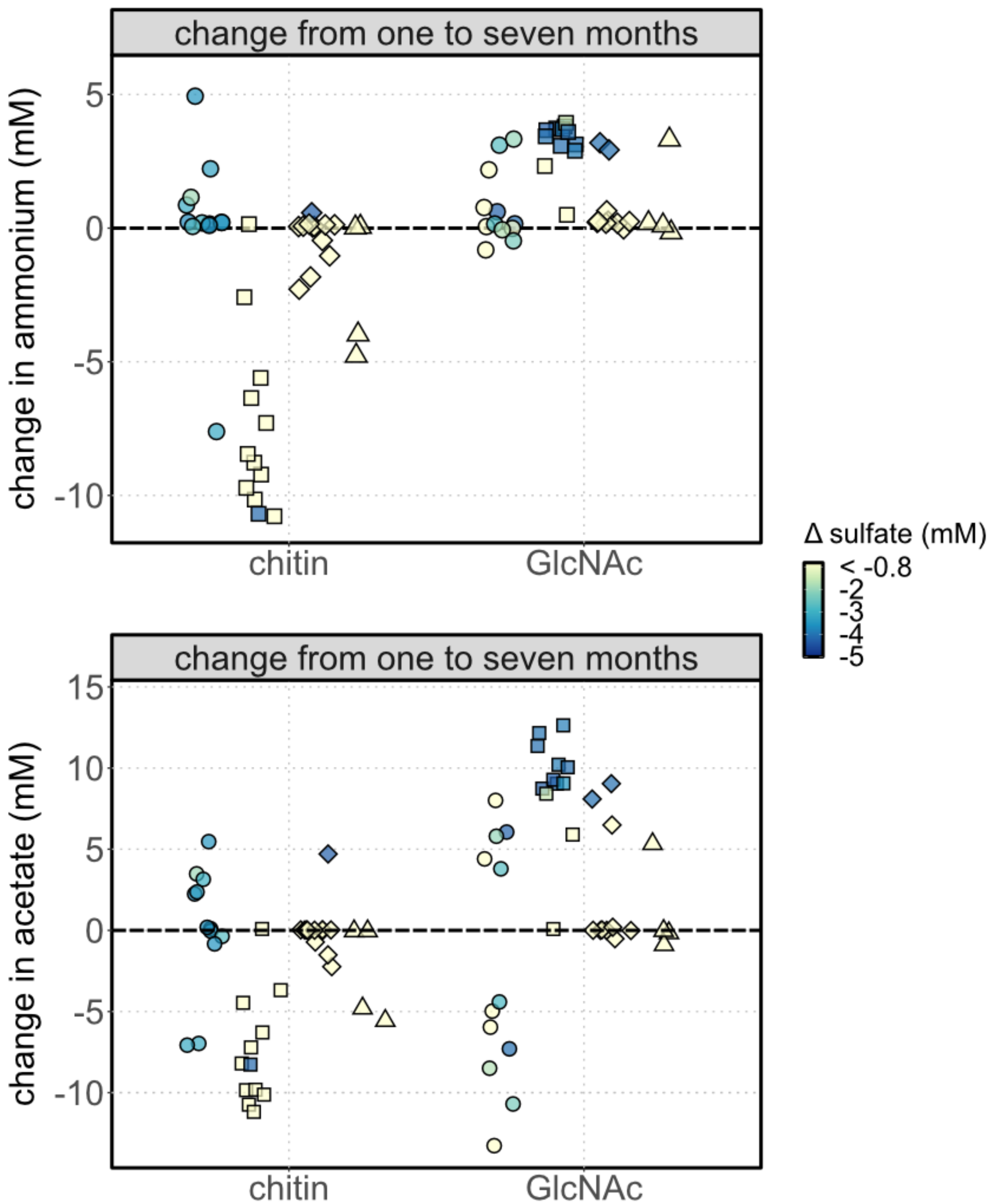

45

FIG S4 Difference plot of acetate and ammonium concentration illustrates the change in metabolite production/consumption over the course of the experiment, from one to seven months. Different dilution treatments are represented by shape. The horizontal line represents no change from one to

seven months, and shading represents differential sulfate concentration over that same span.

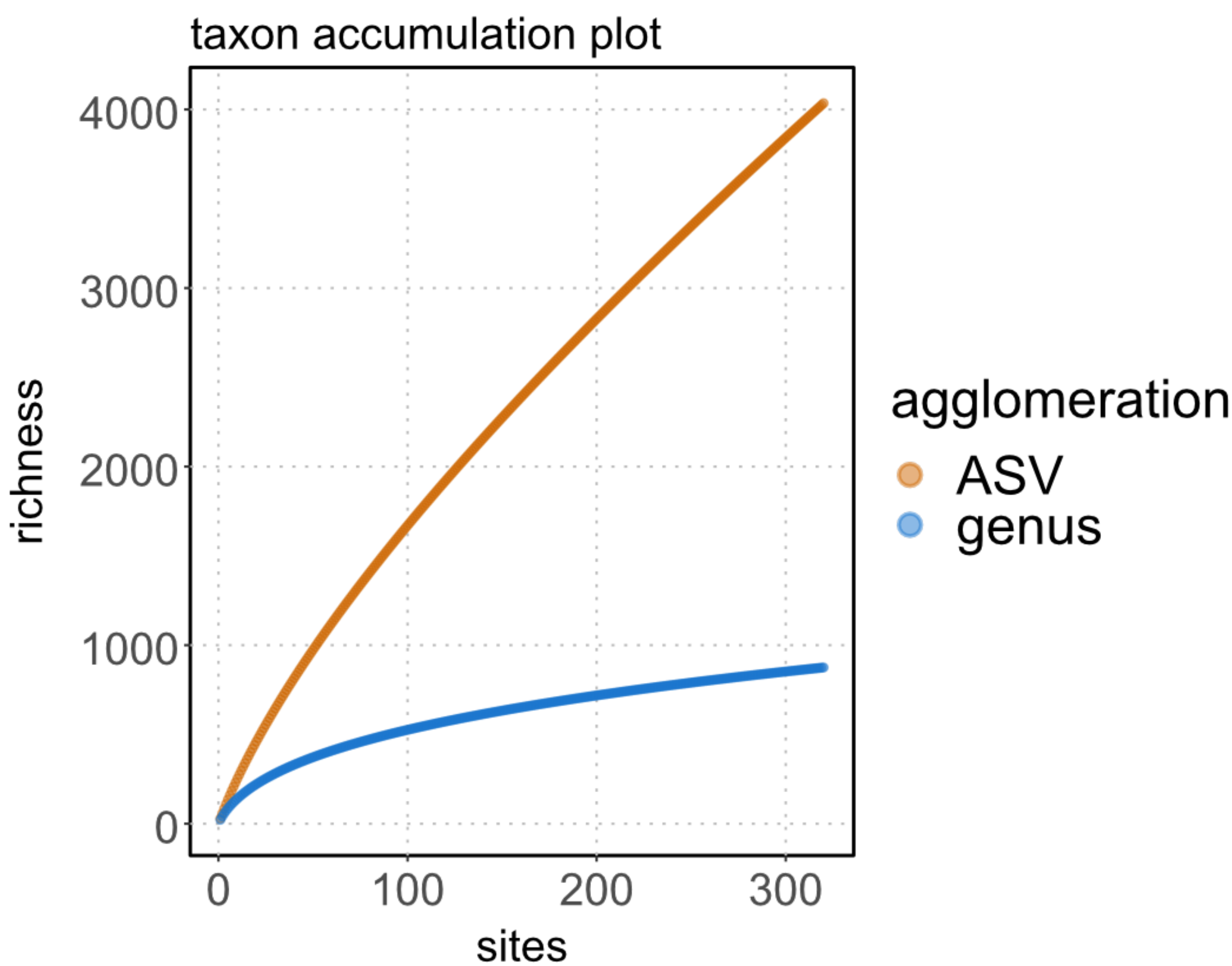

FIG S5 Taxon accumulation plot for 16S sequencing data at the ASV level (orange) and agglomerated by genus (blue).

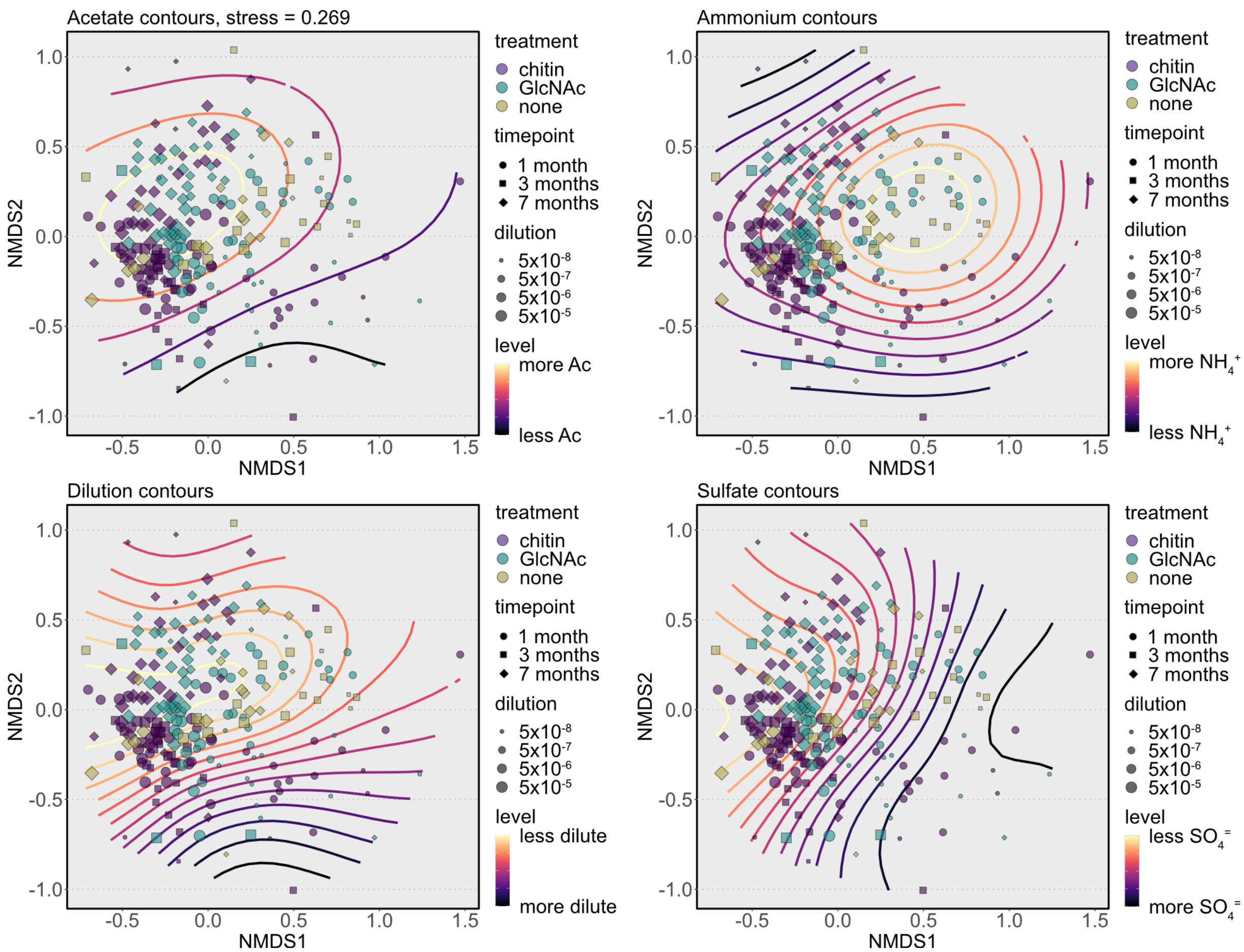

FIG S6 NMDS plot with all samples containing geochemistry data plotted in the same pane. Contours correspond to metabolite concentration (acetate, ammonium, or sulfate) or dilution.

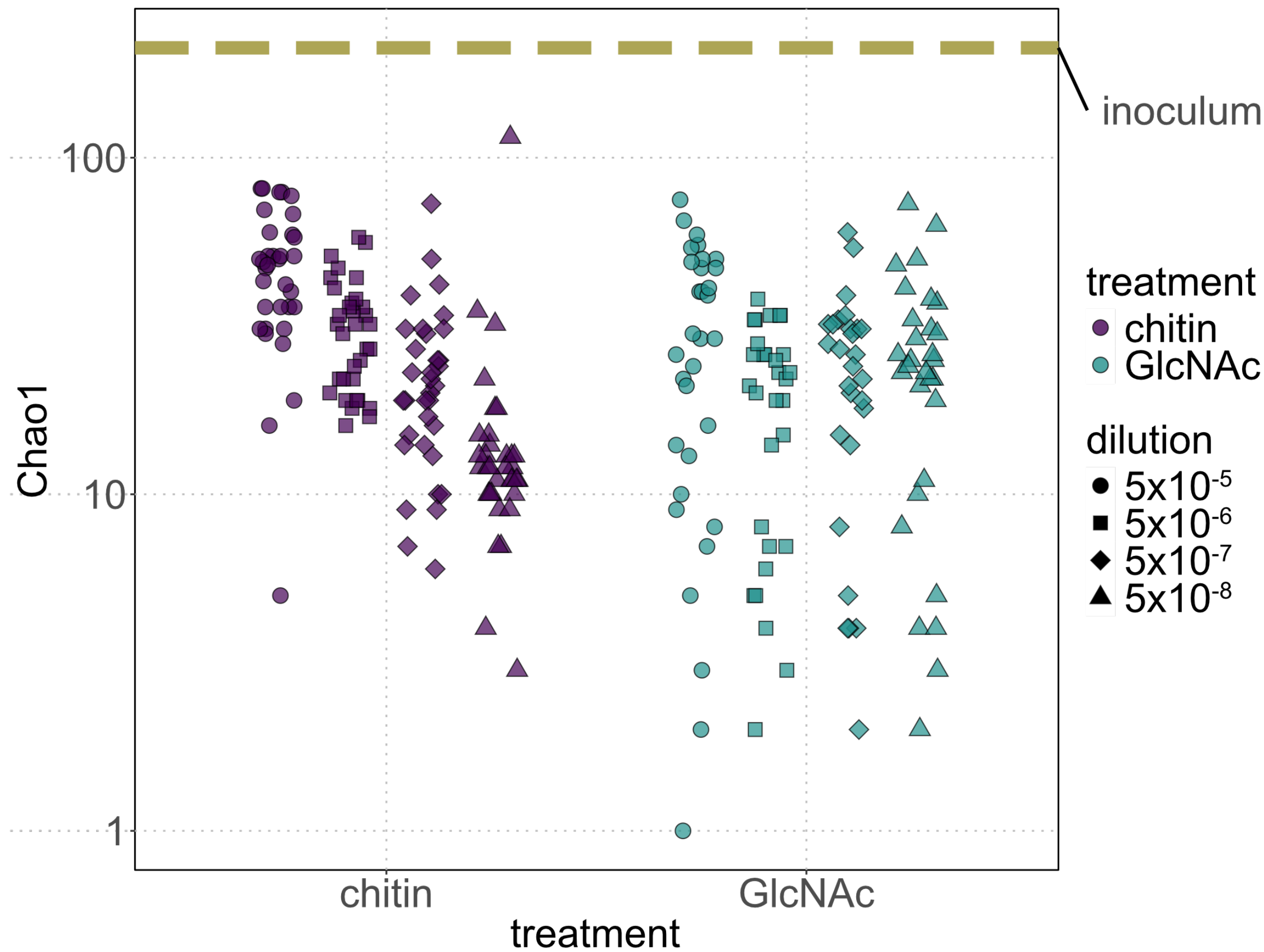

FIG S7 Plot of chitin (purple) and GlcNAc (green) incubation Chao1 richness with source inoculum richness marked with yellow dashed line. While the original inoculum is richer than any of the highly diluted incubations, it remains at the same order of magnitude of richness as the majority of the chitin treatments at all timepoints.

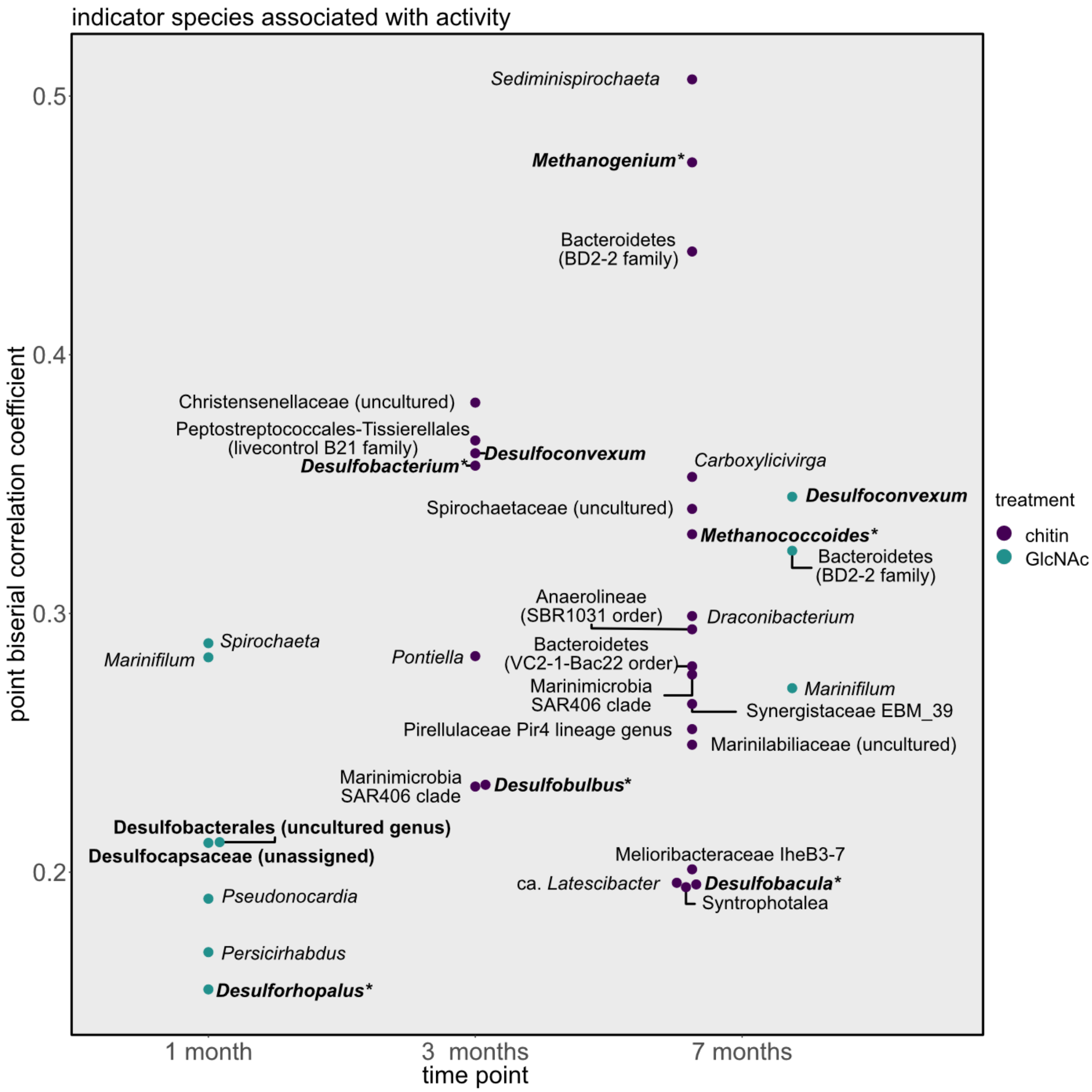

FIG S8 Indicator taxon analysis demonstrates an enhanced number of differentially abundant genera associated with metabolic activity in chitin treatments (purple points) vs GlcNAc treatments (green points). Labels correspond to the lowest taxonomic level description available. Bolded labels indicate putative methanogens or sulfate-reducing bacteria, and asterisks denote genera supported with metabolic gene data (DsrA or McrA).

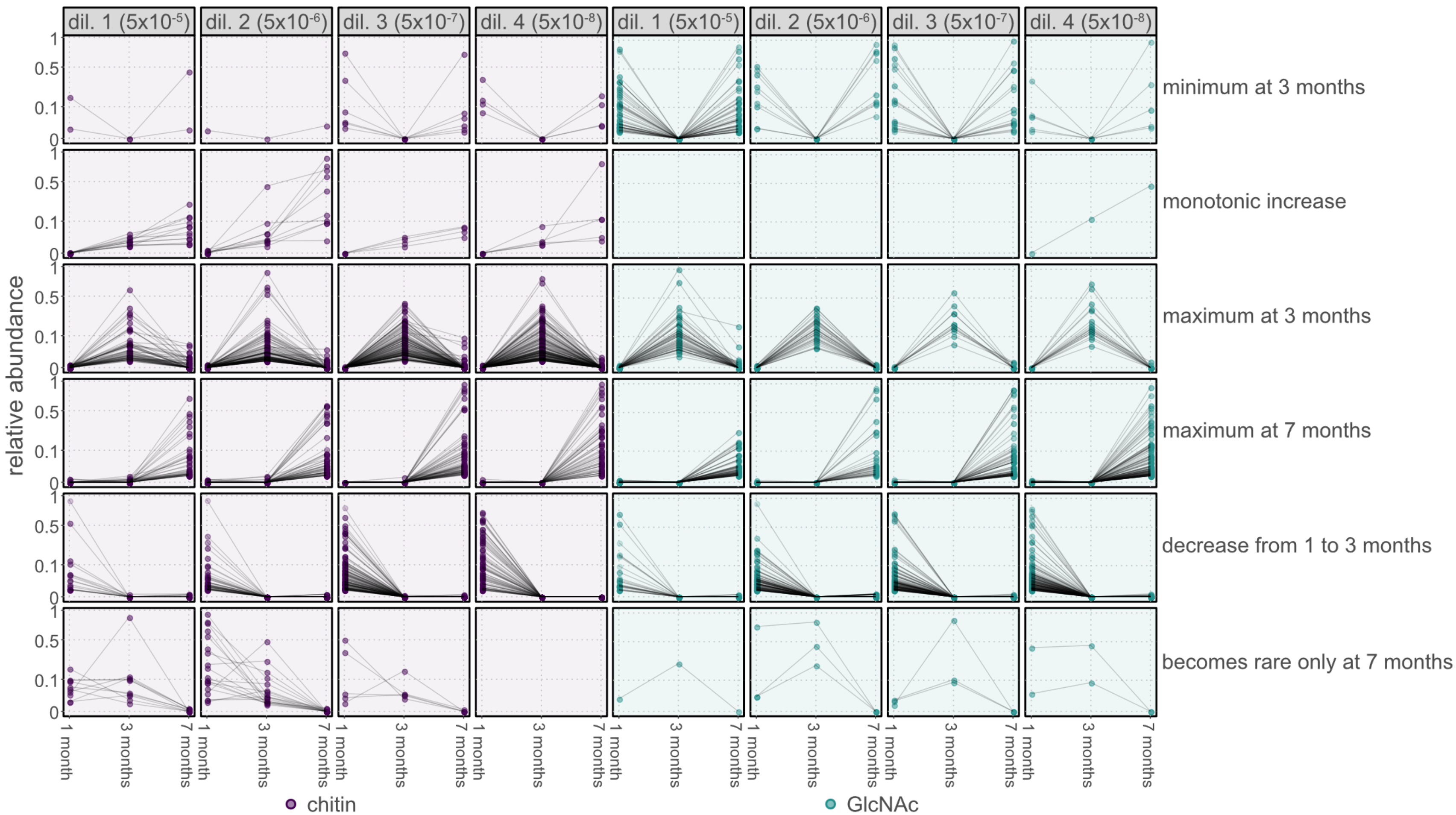

FIG S9. Shifts in rare genus relative abundance over time, separated by general temporal trends (rows). Rare genera here are defined by any genus that was at less than 0.1% relative abundance at any timepoint.

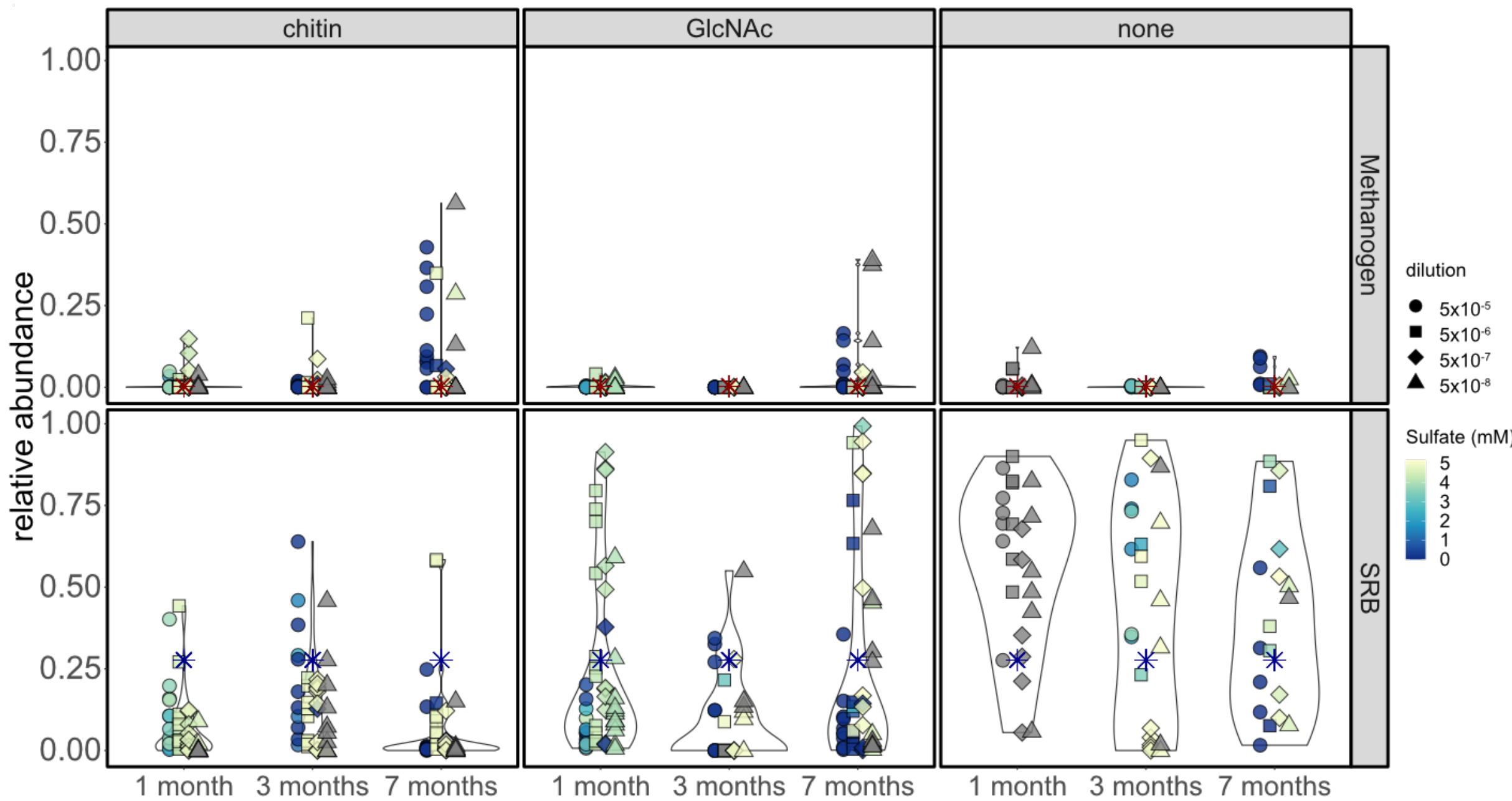

FIG S10 Relative abundance of inferred methanogenic (top) and sulfate-reducing (bottom) taxa with 16S sequencing data. Point fill corresponds to sulfate concentration within each replicate and shape corresponds to dilution. Asterisks correspond to total relative abundance of these taxa in the source sediment.

51

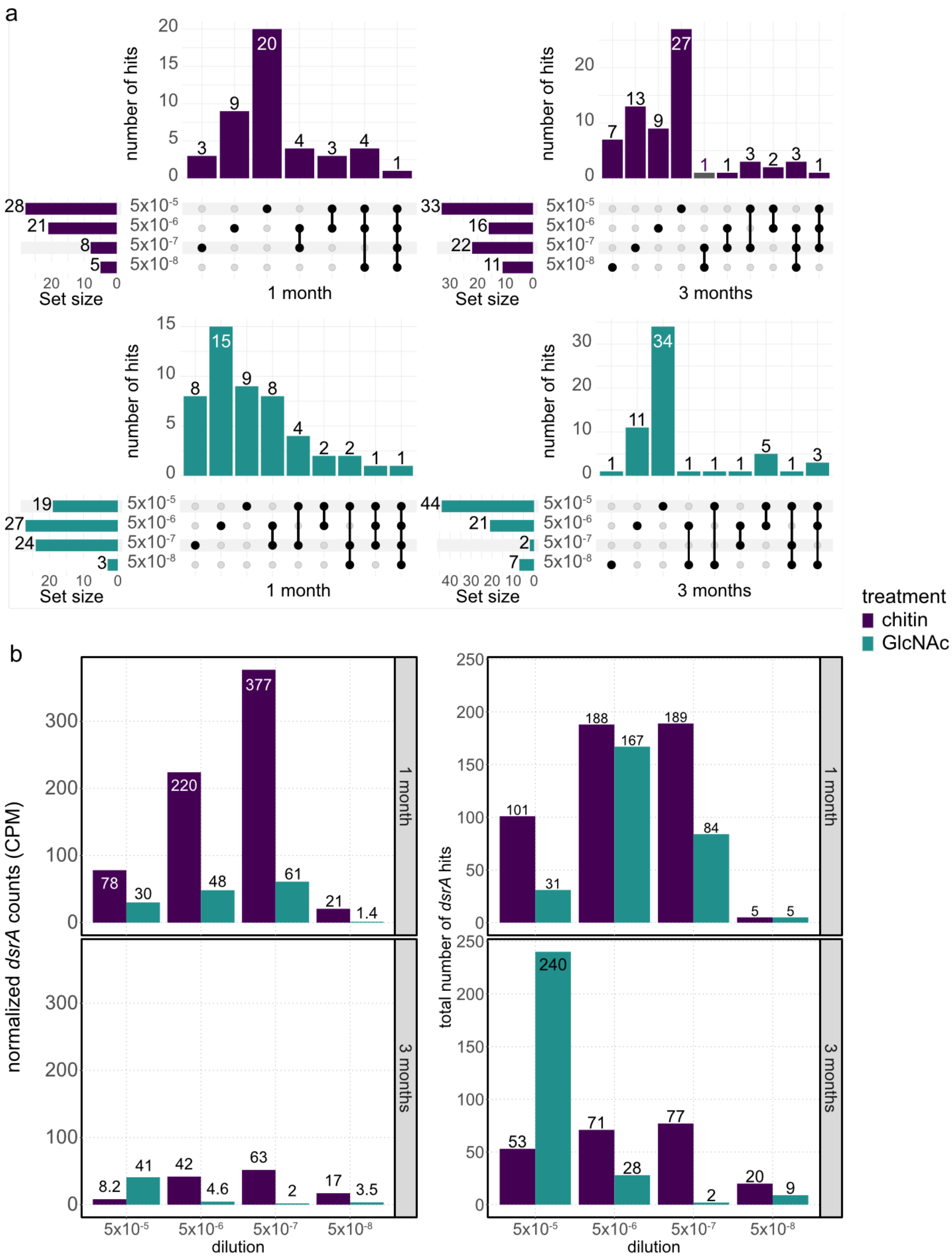
a
number of hits
Set size
1 month
3 months
5x10-5
5x10-6
5x10-7
5x10-8
b
normalized dsrA counts (CPM)
total number of dsrA hits
dilution
1 month
3 months
treatment
chitin
GlcNAc

FIG S11 (a) Sulfite reductase genes (*dsrA*) predominantly correspond to taxonomically unique hits within each dilution. Rows and bar color indicate supplied electron donor; columns indicate incubation time. Dot plots indicate combinations of dilutions with shared hits. Set sizes correspond to the number of unique hits per dilution. (b) Total number of *dsrA* genes found in metagenomic sequences (right) and normalized by read number (counts per million) (left). With the exception of the lowest dilution at the second timepoint, chitin treatments contain more *dsrA* genes per read number than GlcNAc.

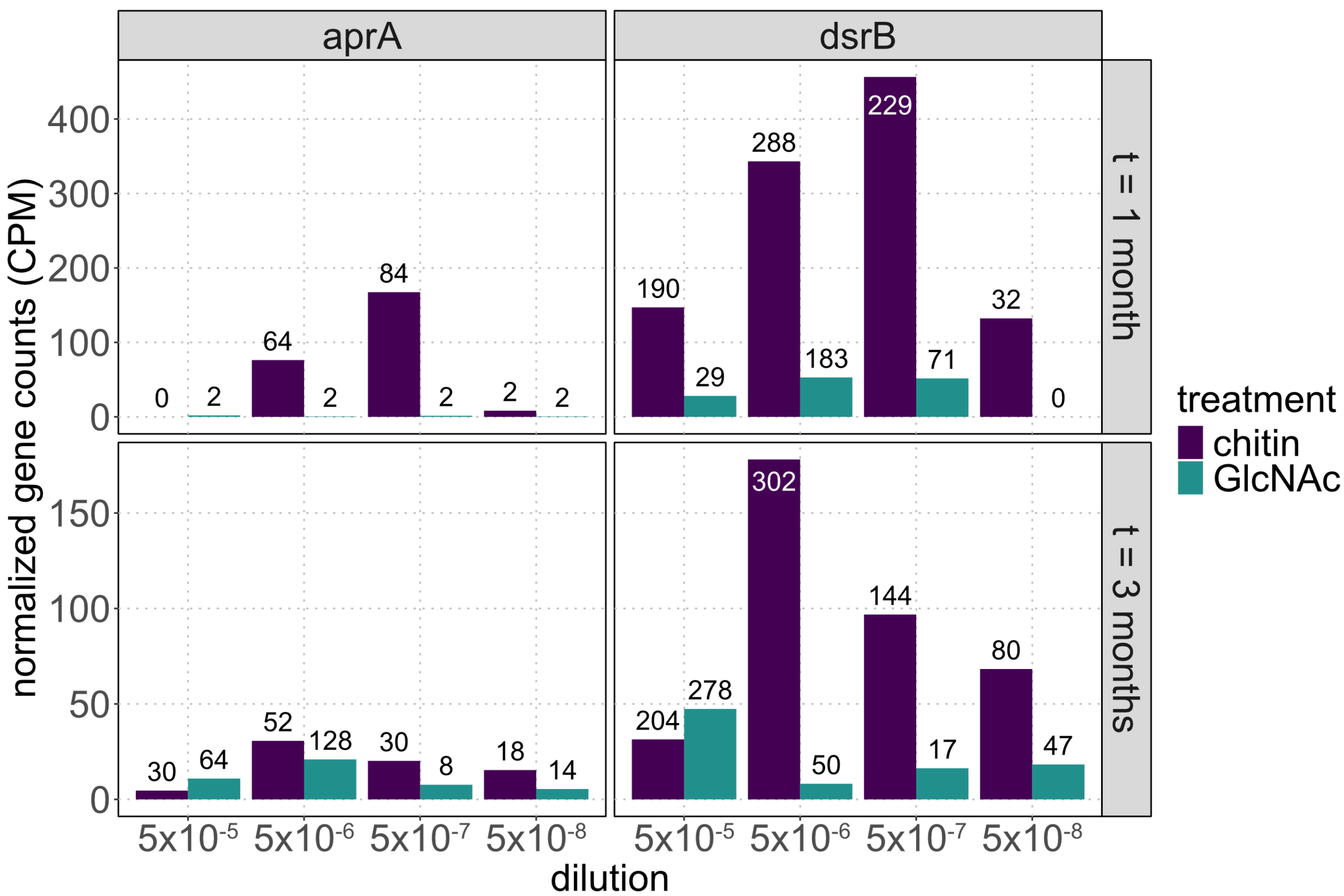

FIG S12 Other sulfate reduction pathway genes adenosine 5′-phosphosulfate reductase (*aprA*; left) and sulfite reductase subunit B (*dsrB*; right) generally agree in distribution to *dsrA* with respect to treatment. Values are presented as normalized gene counts (per million reads).

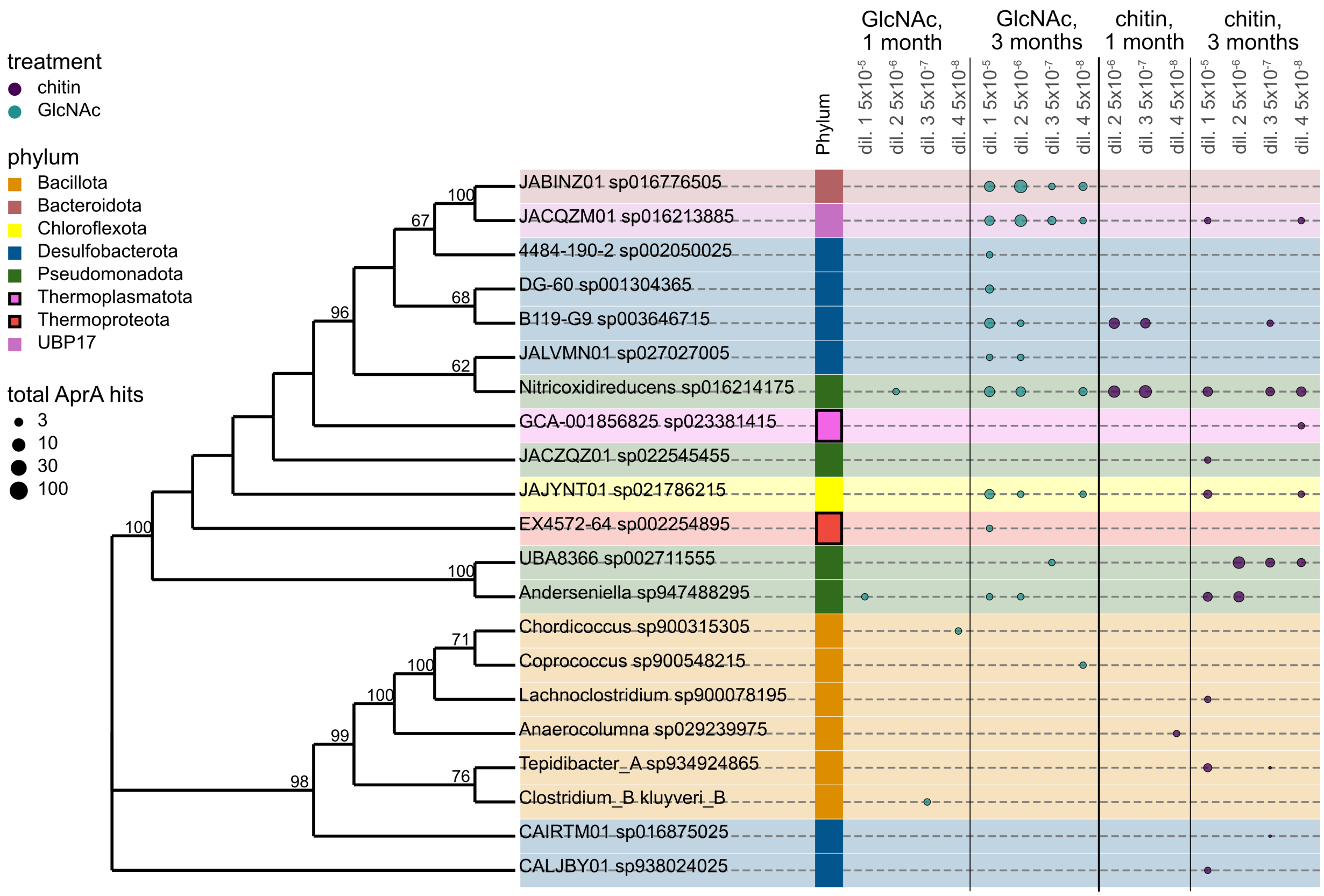

FIG S13 Phylogenetic tree showing the distribution of metagenome sequence hits mined from a GTDB AprA amino acid database. GTDB AprA sequences were aligned with MUSCLE and the tree was generated with IQTREE model LG+I+G4. Node labels correspond to bootstrap support values greater than 50%. Tip labels correspond to species from GTDB while tips and horizontal shading are colored by phylum. The faceted plot to the right contains a summary of the metagenomic sequence distribution per treatment (point color) separated by dilution and time. Point size indicates number of reads corresponding to at least a 95% identity match with the GTDB amino acid database

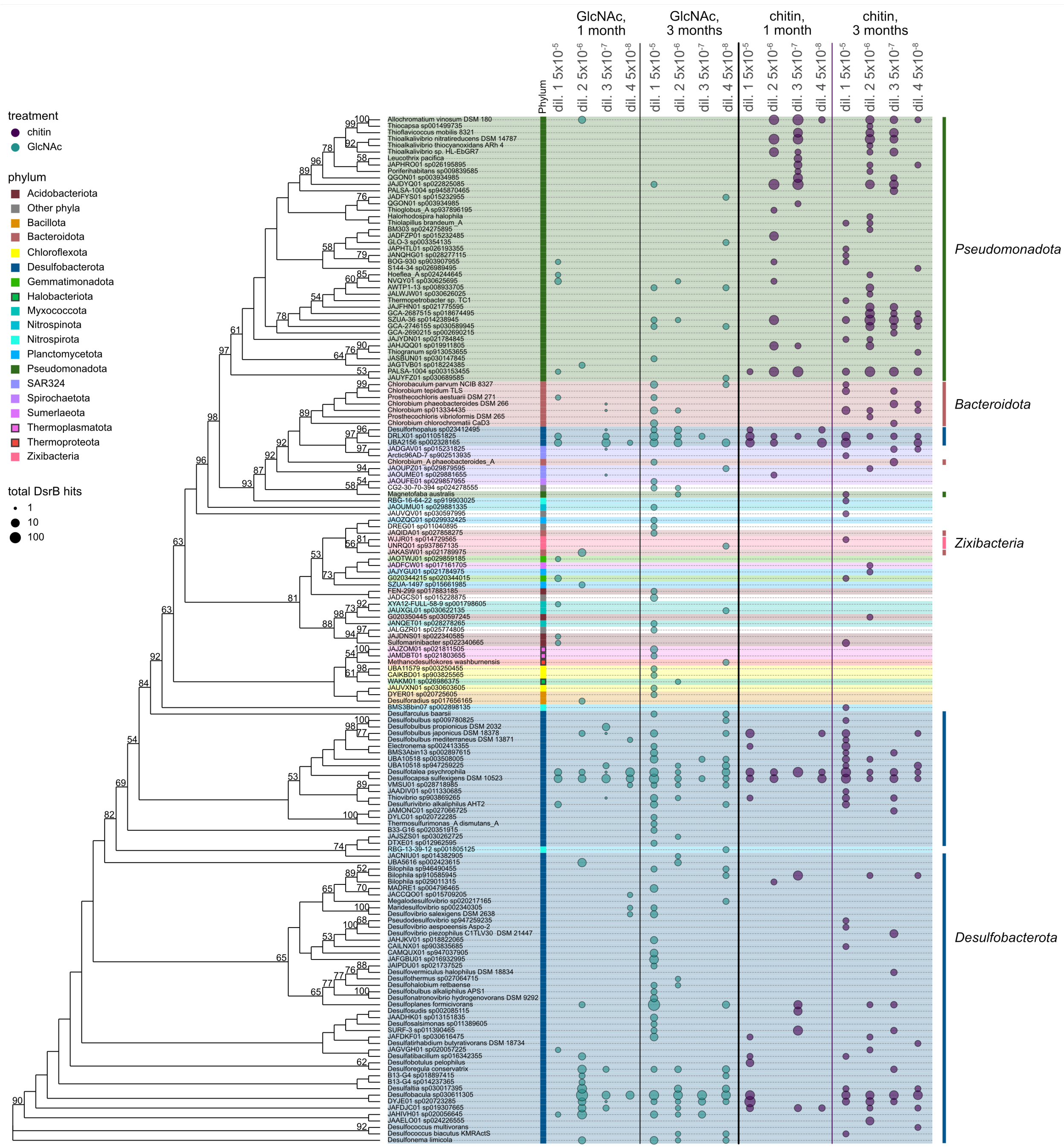

FIG S14 Phylogenetic tree showing the distribution of metagenome sequence hits mined from a GTDB DsrB amino acid database. GTDB DsrB sequences were aligned with MUSCLE and the tree was generated with IQTREE model LG+I+R8. Node labels correspond to bootstrap support values greater than 50%. Tip labels correspond to species from GTDB while tips and horizontal shading are colored by phylum. The faceted plot to the right contains a summary of the metagenomic sequence distribution per treatment (point color) separated by

dilution and time. Point size indicates number of reads corresponding to at least a 95% identity match with the GTDB amino acid database.

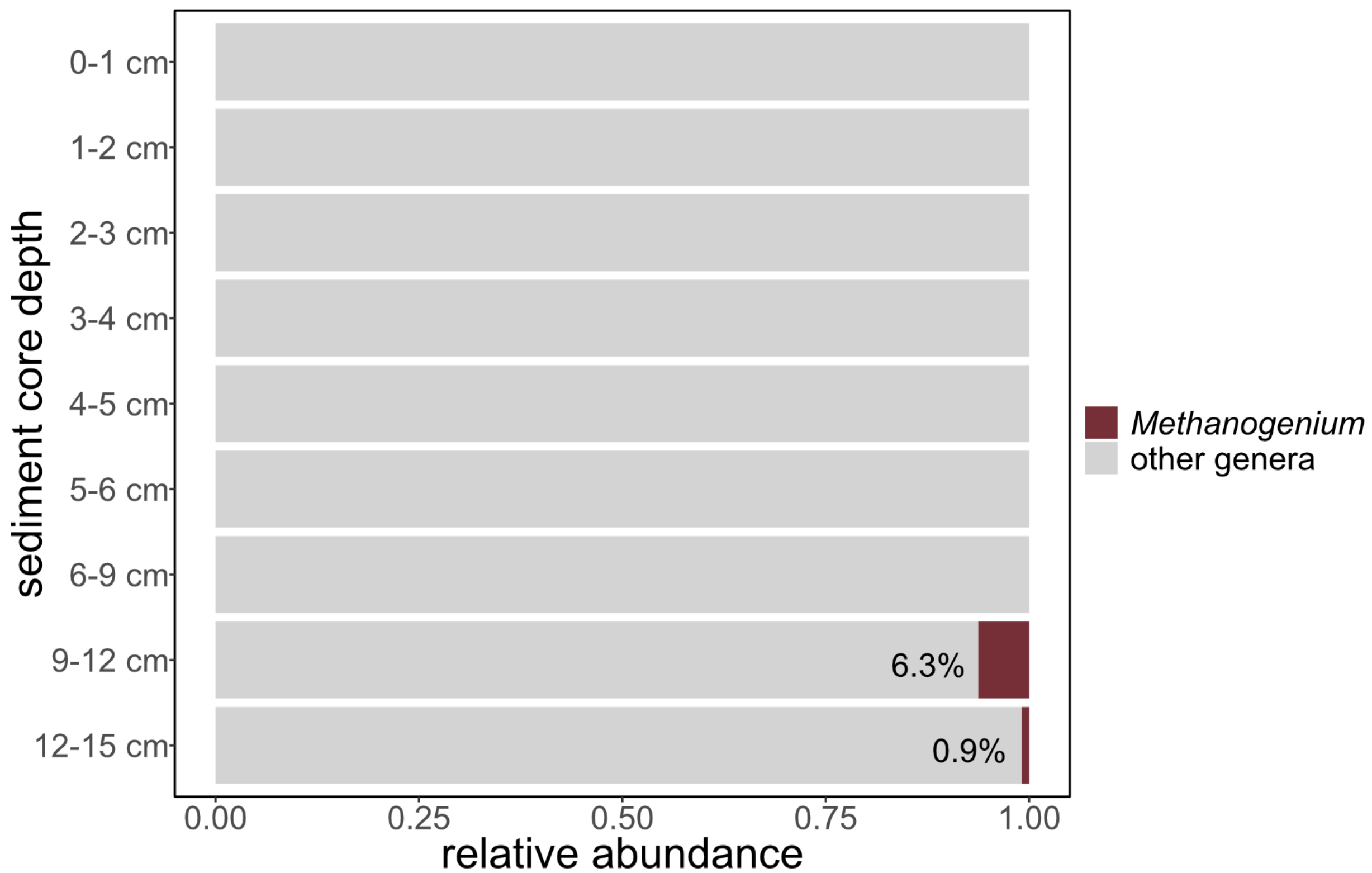

FIG S15 Relative abundance of *Methanogenium* within the inoculum sediment core as determined by 16S rRNA gene amplicon sequencing. The inoculum of the experiment was the 6-9 cm horizon, which contained no detectable *Methanogenium*; however, this genus was found in deeper horizons of the core.

	**1 month**	**3 months**	**7 months**
All incubations	0.241**	0.276**	0.167**
Active incubations	0.1875**	0.611**	*-0.035*

TABLE S1 Per-treatment dissimilarity metric (ANOSIM R statistic) shows a maximum dissimilarity between chitin and GlcNAc treatments at three months in active incubations. Significant values are marked (** $p<0.001$; italics are not statistically significant).

Dil., Months	Chitin					GlcNAc				
	μ_{Obs}	σ_{Obs}	μ_{BP}	σ_{BP}	n	μ_{Obs}	σ_{Obs}	μ_{BP}	σ_{BP}	n
Dil. 1 5x10^{-5}, 1 mo.	40.25	15.65	0.64	0.19	12	29	11.39	0.53	0.22	12
Dil. 1 5x10^{-5}, 3 mos.	57.36	22.44	0.38	0.19	11	6.44	4	0.49	0.31	9
Dil. 1 5x10^{-5}, 7 mos.	46	16.69	0.55	0.12	11	52.18	11.1	0.5	0.22	11
Dil. 2 5x10^{-6}, 1 mo.	36.5	12.83	0.65	0.21	12	25.36	8.54	0.56	0.27	11
Dil. 2 5x10^{-6}, 3 mos.	33.4	10.82	0.43	0.24	10	5	2	0.44	0.19	8
Dil. 2 5x10^{-6}, 7 mos.	23.64	6.41	0.55	0.16	11	24.27	7.02	0.66	0.21	11
Dil. 3 5x10^{-7}, 1 mo.	22.33	11.39	0.44	0.18	12	26.58	11.74	0.58	0.21	12
Dil. 3 5x10^{-7}, 3 mos.	23.36	19.06	0.31	0.1	11	3.8	1.1	0.54	0.21	5
Dil. 3 5x10^{-7}, 7 mos.	23.82	9.6	0.62	0.21	11	30	12.16	0.62	0.24	11
Dil. 4 5x10^{-8}, 1 mo.	12.83	6.71	0.41	0.15	12	27.17	9.31	0.53	0.16	12
Dil. 4 5x10^{-8}, 3 mos.	22.64	31.78	0.33	0.22	11	4.33	2.07	0.49	0.16	6
Dil. 4 5x10^{-8}, 7 mos.	12.73	3.47	0.56	0.23	11	35.33	18.64	0.52	0.25	12

TABLE S2. Means and standard deviations of per-dilution diversity calculated from 16S rRNA gene amplicon sequence data. OBS, observed ASVs; BP, Berger-Parker dominance. Samples with low read abundance were excluded from all analyses as described in Methods.

**Treatment**	**timepoint**	R^2	**p**
GlcNAc	1 month	0.017	0.389
GlcNAc	3 months	0.092	0.182
GlcNAc	7 months	0.041	0.228
chitin	1 month	0.205	3.36E-03
chitin	3 months	0.452	9.8E-03
chitin	7 months	0.51	1E-06

TABLE S3 Sulfate concentration does not correlate strongly with diversity (richness). The table lists multiple linear regression analysis with R^2 and p values reported for each treatment/timepoint pair using model Chao1$\sim$[Sulfate].

**Treatment**	**timepoint**	**dilution**	**read pairs**
GlcNAc	1 month	Dil. 4 $5x10^{-8}$	3592159
GlcNAc	1 month	Dil. 3 $5x10^{-7}$	1375302
GlcNAc	1 month	Dil. 2 $5x10^{-6}$	3455332
GlcNAc	1 month	Dil. 1 $5x10^{-5}$	1029520
chitin	1 month	Dil. 4 $5x10^{-8}$	241919
chitin	1 month	Dil. 3 $5x10^{-7}$	501605
chitin	1 month	Dil. 2 $5x10^{-6}$	839200
chitin	1 month	Dil. 1 $5x10^{-5}$	1292286
GlcNAc	3 months	Dil. 4 $5x10^{-8}$	2570652
GlcNAc	3 months	Dil. 3 $5x10^{-7}$	1041757
GlcNAc	3 months	Dil. 2 $5x10^{-6}$	6111965
GlcNAc	3 months	Dil. 1 $5x10^{-5}$	5867487
chitin	3 months	Dil. 4 $5x10^{-8}$	1171562
chitin	3 months	Dil. 3 $5x10^{-7}$	1487207
chitin	3 months	Dil. 2 $5x10^{-6}$	1696132
chitin	3 months	Dil. 1 $5x10^{-5}$	6491622

TABLE S4 Read output per sample from metagenomic sequencing on HiSeq 2500.

Bin name	total length	# contigs	N50	GC content	% completeness	% redundancy
chitin-01	3420031	117	51395	43.21	100	1.41
chitin-02	3470621	209	25846	42.48	100	2.82
chitin-03	4536287	169	44261	50.38	100	2.82
chitin-04	6259831	138	83848	35.11	100	5.63
chitin-05	7478586	465	23658	37.5	98.59	4.23
chitin-06	3006360	342	11875	44.75	92.96	5.63
chitin-07	4841039	580	10652	42.25	80.28	5.63
chitin-08	1054520	265	3824	33.66	66.2	2.82
chitin-09	1426982	367	3785	35.95	56.34	0
chitin-10	3057467	580	5651	45.78	50.7	1.41
chitin-11	1355109	412	3229	42.96	49.3	2.82
chitin-12	2966155	693	4214	38.03	39.44	2.82
glcnac-01	7002662	92	143043	44.39	100	4.23
glcnac-02	3449119	99	55676	50.03	100	4.23
glcnac-03	3918870	233	26017	40.36	100	5.63
glcnac-04	7665877	341	33494	47.17	95.77	1.41
glcnac-05	4824704	131	69831	52.03	94.37	1.41
glcnac-06	4709244	487	13352	52.49	91.55	1.41
glcnac-07	4785544	221	39516	50.81	92.96	5.63
glcnac-08	1316442	276	5186	31.74	61.97	0
glcnac-09	779072	183	4325	40.54	60.56	0
glcnac-10	3300648	672	5310	47.62	54.93	2.82
glcnac-11	2257557	567	3915	39.32	45.07	1.41

TABLE S5 Summary of bin output merged across samples demonstrates few bins. Bins were generated using Anvi'o v7 [1]. Bin name includes treatment (chitin, GlcNAc) and bin number.

Supplemental text

Indicator taxon analysis of active incubations

Given the strong influence of carbon degradation activity on community dissimilarity, we analyzed the effect of metabolic activity on indicator taxa [2] by binning samples based on activity (e.g., accumulation of at least 0.5 mM acetate or ammonium, or at most 4.2 mM sulfate remaining) and determined the taxa significantly associated ($p<0.05$) with active replicates for each treatment and timepoint. Over twice the number of taxa were associated with active microcosms from the chitin treatments (24) than those incubated with the monomer GlcNAc (10). Taxa associated with activity differed between the one-, three-, and seven-month timepoints, as well (Fig. S7). Seven genera were identified as highly correlated with active GlcNAc treatments at the one-month timepoint, including potential sulfate-reducing taxa *Desulfocapsaceae* and *Desulforhopalus*, and putative saccharide utilizers *Spirochaeta* and *Marinifilum* [3, 4]. No indicator taxa were identified at the three-month timepoint in these active GlcNAc treatments, and at the seven-month timepoint, substantially fewer indicator genera were identified versus the first timepoint. Only three genera were associated with the final timepoint: *Desulfoconvexum*, a member of the Bacteroidetes BD2-2 family, and *Marinifilum*. This trend differed from the chitin treatments, which did not have any identified indicator taxa at the first timepoint, and seven identified in incubations after three months. These included putative sulfate reducers *Desulfobacterium* and *Desulfoconvexum*, the potential saccharide-utilizing *Firmicutes Christensenellaceae* and *Peptostreptococcales-Tissierellales* [5, 6], and the putative sulfonated saccharide-utilizing *Verrucomicrobia*, *Pontiella* [7] The member of the *Peptostreptococcales-Tissierellales* belonged to an uncultured cold seep-associated genus that branched closely with the disaccharide-utilizing genus *Geosporobacter* [5]. At the final, seven-month timepoint, there were 17 taxa associated with active chitin-amended treatments (Fig. S7). The taxa that were most associated with activity included putative heterotrophs *Sediminispirochaeta*, a member of the *Bacteroidetes* BD2-2 family, *Carboxylicivirga*, and an uncultured member of the *Spirochaetaceae*, and, notably, the methanogenic genera *Methanogenium* and *Methanococcoides*. Of the predicted sulfate-reducing taxa, *Desulfobacterium* species are known to be capable of acetate

oxidation [8], while *Desulforhopalus* and *Desulfoconvexum* are both considered incomplete oxidizers using fatty acids or primary alcohols rather than acetate [9, 10]. Moreover, the two methanogen genera associated with the final timepoint from active chitin microcosms were expected to have differing niches and were not observed in GlcNAc microcosms. While *Methanococcoides* was associated with noncompetitive methylotrophic substrates and thus expected to potentially be coincident with sulfate reducers, *Methanogenium* was thought to be hydrogenotrophic [11, 12, 13].

Supplemental methods

Sample collection, incubation conditions, and media design

The sediment core was collected in December 2018 on research cruise WF12-18 on the R/V *Western Flyer* with ROV *Doc Ricketts* at GPS coordinates 36.769873 N, 122.0829 W and 1018 m depth. The collection site was at a former whale fall site (emplacement October 2004) in Monterey Bay at 1018 m depth. Depth horizons were sectioned from the sediment core and the 6-9 cm horizon was used for inoculum after <1 year anaerobic storage at 4 °C in an argon-gassed Mylar bag. Colloidal chitin was prepared by dissolving 25 g chitin (Alfa Aesar catalog number J61206-36) in 150 mL 12N HCl; the resulting slurry was strained through a cheesecloth and precipitated in 1 L ice cold milli-Q water. After an overnight incubation at 4 °C, the mixture was neutralized to pH 7 with sodium hydroxide and washed three times with milli-Q water using a 4 °C centrifugation step (10,000 x *g*, 20 minutes). The washed colloidal chitin was then freeze-dried before use in incubations. The sulfate-free artificial seawater medium consisted of 410.7 mM NaCl, 52.5 mM $MgCl_2$, 8.9 mM $CaCl_2$, 8.9 mM KCl, 0.84 mM KBr, 0.44 mM H_3BO_3, 0.1 mM $SrCl_2$, 0.07 mM NaF, 0.055 mM K_2HPO_4, 0.022 mM KH_2PO_4, 25 mM HEPES, and 1x SL-11 trace elements at pH 6 (15.467 µM Na_2-EDTA pH 7, 7.545 µM $FeCl_2$, 0.514 µM $ZnCl_2$, 0.505 µM $MnCl_2$, 0.097 µM H_3BO_3, 1.463 µM $CoCl_2$, 0.012 µM $CuCl_2$, 0.185 µM $NiCl_2$, 0.149 µM Na_2MoO_4). The medium was pH-adjusted to 7.5. The sulfate-free artificial seawater medium was supplemented with 5 mM sodium sulfate and, depending on treatment, colloidal chitin (5.7 mg/mL), N-acetylglucosamine (5 mM), or no electron donor. Tenfold serial dilutions of a 1:1 sediment slurry in 1.75 mL total volume were anaerobically prepared in 2 mL

deep well 96-well plates (VWR part number 89237-526) with plate rows corresponding to dilution and plate columns corresponding to biological replicates. These deep well plates were capped with silicone mats (VWR part number 89237-538) and sealed in separate Mylar bags with an anoxic headspace for each treatment. After incubation at 10 °C (at ambient pressure) for one, three, and seven months, each well was subsampled for microscopy, metagenome sequencing, 16S rRNA gene amplicon sequencing, and geochemistry. At each timepoint, 300 µL sample was filtered through a 0.2-micron filter plate (Pall product number 8019) into a polystyrene storage plate, which was then sealed with an adhesive film (Bio-Rad Microseal B) for ion chromatography. The filtrand was saved at -80 °C for DNA extraction. 200 µL of sample at each timepoint was fixed with a final concentration of 1% paraformaldehyde and stored at 4 °C for microscopy.

Geochemistry: quantification of major ions and metabolites

Samples for ion chromatography were stored at -20 °C until analysis, when they were diluted 1:50 in Type 1 ultrapure water and run on Dionex ICS-2000 or Thermo Integrion HPIC as previously described [14] on an AS19 anions and CS16 cations column. Analysis was focused on all twelve replicates from dilutions $5x10^{-5}$ to $5x10^{-7}$, with four replicates from the highest dilution ($5x10^{-8}$) for a total of 40 samples analyzed at each timepoint with some exceptions. Six samples were removed from GlcNAc treatments at the first and second timepoints (first timepoint: two samples at $5x10^{-7}$; second timepoint: three samples at $5x10^{-6}$ and one sample at $5x10^{-7}$) due to lack of sample; at the first timepoint, all twelve replicates from this treatment at the highest dilution ($5x10^{-8}$) were analyzed. Samples were run at the Water and Environment Laboratory (Caltech).

DNA extraction and sequencing

Samples were extracted using the Beckman DNAdvance kit (Beckman product number A48705) using the following protocol (adapted from [15]). Samples were lysed with 20 µL of a 1,000U/µL lysozyme solution at 37 °C for 1.5 hours, 140 µL of a Proteinase K lysis mixture (132.25 µL LBH lysis buffer, 5.25 µL 0.8 U/µL Proteinase K, 2.5 µL freshly prepared 1M dithiothreitol) at 55 °C for 16-20 hours, and 40 µL of a 10% SDS solution at 65 °C for 2 hours. Lysate was gently mixed with 50 µL PBBA pre-bind buffer, avoiding

bubbles, followed by addition of 85 µL BBE bind buffer. After room temperature incubation for 1 minute, beads were separated with a 7-minute incubation on a magnetic plate stand. Supernatant was removed and beads were washed three times with 170 µL of a freshly made 70% ethanol solution. Each wash involved thorough resuspension of pellet and subsequent incubation on a magnetic plate stand until solution cleared. After the final wash, remaining supernatant was removed using a 10 µL multichannel pipette and beads were incubated at room temperature for 5 minutes to allow drying of residual ethanol. The plate was then removed from magnet and DNA was eluted with a resuspension of beads in 30 µL EBA elution buffer, then magnetic separation and transfer of eluted DNA 10 µL at a time into a storage plate. Extracted DNA was quantified with Picogreen (Invitrogen product number P7589), according to manufacturer's protocol. Samples were submitted for 16S rRNA gene sequencing of the V4 hypervariable region to Argonne National Laboratories (Earth Microbiome Project barcoded primer set 515F 5'-GTGYCAGCMGCCGCGGTAA/806R 5'-GGACTACNVGGGTWTCTAAT) or Laragen (515F 5'-GTGYCAGCMGCCGCGGTAA/926R 5'-CCGYCAATTYMTTTRAG TTT). Both primer sets had Illumina adapters appended to the 5' end as described previously [14, 16]. Samples were submitted for metagenome sequencing to the MIT core sequencing facility (Cambridge, MA, USA) on a HiSeq 2500 (Illumina) after library preparation using the Nextera XT library preparation kit and quality control with a TapeStation 4200 (Agilent) using the HSDNA D1000 screen tape, both according to manufacturer's protocol.

16S rRNA amplicon sequence analysis

Demultiplexed 16S amplicon reads were put through the QIIME2 pipeline with denoised ASV abundances generated using DADA2 [17, 18]. Taxonomy classification was done using a classifier trained on the SILVA SSU database release 138 [19, 20]. Samples with fewer than 20 reads were removed from the analysis. All R analyses were done using R Statistical Software (version 4.0.3). Decontamination and abundance visualization of 16S data was done using the R phyloseq and decontam packages [21, 22]. Diversity, dissimilarity, and species accumulation analyses were done using the vegan package [23] with NMDS analysis done on fourth-root transformed genus-agglomerated relative abundance data. Indicator species analysis was done using the indicspecies package [24]

with the point biserial correlation coefficient corrected for site number. Correlation analysis was done using fastspar [25]. Visualization of networks was done using the igraph R package [26] and Cytoscape [27]. Correlation networks were then clustered with MCODE [28]. Nestedness was demonstrated through Euler plots of presence-absence transformed 16S rRNA sequence data with all dilutions and replicates combined for each treatment at each timepoint. Sulfate reducing and methanogenic taxa were defined generally as members of the phylum Desulfobacterota or orders Methanosarcinales, Methanofastidiosales (formerly the WSA2 group [29]) and Methanomicrobiales, respectively (Supp. Files).

Metagenomic analysis of metabolic marker genes

Metabolic marker genes for sulfate reduction (*dsrA*) and methanogenesis (*mcrA*) were mined from metagenomic sequence reads using a previously described protocol [30] and manually curated databases derived from GTDB release 202 (Supp. Files). To prepare a reliable Hidden Markov Model (Eddy, 1998) for searching GTDB, taxonomically diverse DsrA and McrA amino acid sequences were identified from a non-redundant set of bacterial and archaeal genomes in the IMG database [31] using the BLASTP [32] function on their website. These sequences were filtered using UCLUST [33] to extract sequences that were at least 50% dissimilar in sequence identity. The remaining highly dissimilar sequences were used to construct an HMM using the HMMbuild function of HMMer v.3.1b2 [34]. These HMMs were then used to query the genomes from GTDB r202 by using the hmmsearch function of HMMer. The resultant databases are made available as part of the supplement. Metagenome reads were searched against the database using DIAMOND [35]. These reads were then searched against an outgroup consisting of the NCBI-nr protein reference database, and the BLAST score ratio (BSR) calculated between the two searches; a BSR threshold of 0.75 was applied. Reference DsrA phylogenetic trees were built using a MUSCLE alignment [36] of the database sequences corresponding to mined metagenome sequences matching at least 95% identity with IQTREE [37] model LG+R6, bootstrapped 100 times. Reference McrA phylogenetic trees were generated similarly, with IQTREE model LG+F+G4. Visualization of trees was done with iTOL [38]. Metagenome sequence reads were used rather than assembled bins due to the low

number of quality bins (Table S5). These bins were generated manually after coassembly with SPADES v3.15.2 [39] in Anvi'o v7 [1]. Preliminary coassembly to generate chitin and GlcNAc metabolic pathway gene data (Data S6) was additionally done using Plass [40] to coassemble metagenomic data by combining all dilutions at each treatment and timepoint, clustering using MMseqs [41] at 90% sequence identity over 90% of the length of the longer protein product, conducting profile Hidden Markov Model searches for metabolic genes on assembled contigs using hmmsearch [34, 42], and mapping forward reads onto Plass contigs using MMseqs with default settings [41] to get abundance data for each sample. The abundance data were converted into effective gene diversity by calculating $e^{Shannon}$. SingleM 0.20.3 was used to extract taxonomy data from metagenomic reads using the default settings [43]. These data were compared against the 16S rRNA gene amplicon sequence data for the same sample set to assess the diversity represented in each dataset (Data S7).

Description of supplemental files

Data S1 (xlsx): Similarity Percentage (SIMPER) analysis on significant ANOSIM analyses listing contribution of different genera to dissimilarity between chitin and GlcNAc treatments at each timepoint. Non-statistically significant rows (p>0.05) are italicized.

Data S2 (xlsx): a tabulation of the taxonomy data assigned to the "methanogen" or "SRB" functional groups.

Data S3 (xlsx): a tabulation of the data found in Figure 6, including the total number of MCODE-generated clusters, the total number of clusters containing nodes with methanogenic taxa, and the total number of clusters containing nodes in the Desulfobacterota for each treatment, timepoint, and dilution.

Data S4 (txt): GTDB release 202 amino acid sequences of DsrA pulled using a constructed DsrA HMM.

Data S5 (txt): GTDB release 202 amino acid sequences of McrA pulled using a constructed McrA HMM.

Data S6 (xlsx): Effective gene diversity of genes involved in chitin and GlcNAc metabolism across samples generally show increased diversity in the chitin treatment at the three month timepoint.

Data S7 (xlsx): SingleM output containing relative abundance of taxonomy data present in each sample. 74 total genera were extracted from the metagenomic data with SingleM across the entire dataset, in comparison to 16S rRNA gene amplicon data where there are 134 genera distributed across the same samples.

References

[1] A. Murat Eren, Evan Kiefl, Alon Shaiber, Iva Veseli, Samuel E. Miller, Matthew S. Schechter, Isaac Fink, Jessica N. Pan, Mahmoud Yousef, Emily C. Fogarty, Florian Trigodet, Andrea R. Watson, Özcan C. Esen, Ryan M. Moore, Quentin Clayssen, Michael D. Lee, Veronika Kivenson, Elaina D. Graham, Bryan D. Merrill, Antti Karkman, Daniel Blankenberg, John M. Eppley, Andreas Sjödin, Jarrod J. Scott, Xabier Vázquez-Campos, Luke J. McKay, Elizabeth A. McDaniel, Sarah L. R. Stevens, Rika E. Anderson, Jessika Fuessel, Antonio Fernandez-Guerra, Lois Maignien, Tom O. Delmont, and Amy D. Willis. Community-led, integrated, reproducible multi-omics with anvi'o. *Nature Microbiology*, 6(1):3–6, January 2021. ISSN 2058-5276. doi: 10.1038/s41564-020-00834-3.

[2] Miquel De Cáceres, Pierre Legendre, and Marco Moretti. Improving indicator species analysis by combining groups of sites. *Oikos*, 119(10):1674–1684, October 2010. ISSN 00301299. doi: 10.1111/j.1600-0706.2010.18334.x.

[3] C. S. Harwood and E. Canale-Parola. Spirochaeta isovalerica sp. nov., a Marine Anaerobe That Forms Branched-Chain Fatty Acids as Fermentation Products. *International Journal of Systematic and Evolutionary Microbiology*, 33(3):573–579, 1983. ISSN 1466-5034. doi: 10.1099/00207713-33-3-573.

[4] Hyunsoo Na, Seil Kim, Eun Young Moon, and Jongsik Chun. Marinifilum fragile gen. nov., sp. nov., isolated from tidal flat sediment. *International Journal of Systematic and Evolutionary Microbiology*, 59(9):2241–2246, 2009. ISSN 1466-5034. doi: 10.1099/ijs.0.009027-0.

[5] Nihel Klouche, Marie-Laure Fardeau, Jean-François Lascourrèges, Jean-Luc Cayol, Hocine Hacene, Pierre Thomas, and Michel Magot. Geosporobacter subterraneus gen. nov., sp. nov., a spore-forming bacterium isolated from a deep subsurface aquifer. *International Journal of Systematic and Evolutionary Microbiology*, 57(8): 1757–1761, 2007. ISSN 1466-5034. doi: 10.1099/ijs.0.64642-0.

[6] Xiaoying Liu, Jessica L. Sutter, Jacobo de la Cuesta-Zuluaga, Jillian L. Waters, Nicholas D. Youngblut, and Ruth E. Ley. Reclassification of Catabacter hongkongensis as Christensenella hongkongensis comb. nov. based on whole genome analysis. *International Journal of Systematic and Evolutionary Microbiology*, 71 (4):004774, April 2021. ISSN 1466-5026. doi: 10.1099/ijsem.0.004774.

[7] Daan M. Van Vliet, Yuemei Lin, Nicole J. Bale, Michel Koenen, Laura Villanueva, Alfons J. M. Stams, and Irene Sánchez-Andrea. Pontiella desulfatans gen. nov., sp. nov., and Pontiella sulfatireligans sp. nov., Two Marine Anaerobes of the Pontiellaceae fam. nov. Producing Sulfated Glycosaminoglycan-like Exopolymers. *Microorganisms*, 8(6):920, June 2020. ISSN 2076-2607. doi: 10.3390/microorganisms8060920.

[8] F. Bak and F. Widdel. Anaerobic degradation of indolic compounds by sulfate-reducing enrichment cultures, and description of Desulfobacterium indolicum gen. nov., sp. nov. *Archives of Microbiology*, 146(2):170–176, November 1986. ISSN 1432-072X. doi: 10.1007/BF00402346.

[9] M. F. Isaksen and Andreas Teske. Desulforhopalus vacuolatus gen. nov., sp. nov., a new moderately psychrophilic sulfate-reducing bacterium with gas vacuoles isolated from a temperate estuary. *Archives of Microbiology*, 166(3):160–168, September 1996. ISSN 0302-8933, 1432-072X. doi: 10.1007/s002030050371.

[10] Martin Könneke, Jan Kuever, Alexander Galushko, and Bo Barker Jørgensen. Desulfoconvexum algidum gen. nov., sp. nov., a psychrophilic sulfate-reducing bacterium isolated from a permanently cold marine sediment. *International Journal of Systematic and Evolutionary Microbiology*, 63(Pt_3):959–964, 2013. ISSN 1466-5034. doi: 10.1099/ijs.0.043703-0.

[11] Taiwo S. Akinyemi, Nana Shao, and William B. Whitman. Methanogenium. In *Bergey's Manual of Systematics of Archaea and Bacteria*, pages 1–9. John Wiley & Sons, Ltd, 2019. ISBN 978-1-118-96060-8. doi: 10.1002/9781118960608.gbm00507.pub2.

[12] Stéphane L'Haridon, Morgane Chalopin, Delphine Colombo, and Laurent Toffin. Methanococcoides vulcani sp. nov., a marine methylotrophic methanogen that uses betaine, choline and N,N-dimethylethanolamine for methanogenesis, isolated from a mud volcano, and emended description of the genus Methanococcoides. *International Journal of Systematic and Evolutionary Microbiology*, 64(Pt_6):1978–1983, 2014. ISSN 1466-5034. doi: 10.1099/ijs.0.058289-0.

[13] J. A. Romesser, R. S. Wolfe, F. Mayer, E. Spiess, and A. Walther-Mauruschat. Methanogenium, a new genus of marine methanogenic bacteria, and characterization of Methanogenium cariaci sp. nov. and Methanogenium marisnigri sp. nov. *Archives of Microbiology*, 121(2):147–153, May 1979. ISSN 1432-072X. doi: 10.1007/BF00689979.

[14] Daan R. Speth, Feiqiao B. Yu, Stephanie A. Connon, Sujung Lim, John S. Magyar, Manet E. Peña-Salinas, Stephen R. Quake, and Victoria J. Orphan. Microbial communities of Auka hydrothermal sediments shed light on vent biogeography and the evolutionary history of thermophily. *The ISME Journal*, pages 1–15, March 2022. ISSN 1751-7370. doi: 10.1038/s41396-022-01222-x.

[15] Rachel E. Szabo, Sammy Pontrelli, Jacopo Grilli, Julia A. Schwartzman, Shaul Pollak, Uwe Sauer, and Otto X. Cordero. Historical contingencies and phage induction diversify bacterioplankton communities at the microscale. *Proceedings of the National Academy of Sciences*, 119(30):e2117748119, July 2022. doi: 10.1073/pnas.2117748119.

[16] J. Gregory Caporaso, Christian L. Lauber, William A. Walters, Donna Berg-Lyons, James Huntley, Noah Fierer, Sarah M. Owens, Jason Betley, Louise Fraser, Markus

Bauer, Niall Gormley, Jack A. Gilbert, Geoff Smith, and Rob Knight. Ultra-high-throughput microbial community analysis on the Illumina HiSeq and MiSeq platforms. *The ISME Journal*, 6(8):1621–1624, August 2012. ISSN 1751-7370. doi: 10.1038/ismej.2012.8.

[17] Evan Bolyen, Jai Ram Rideout, Matthew R. Dillon, Nicholas A. Bokulich, Christian C. Abnet, Gabriel A. Al-Ghalith, Harriet Alexander, Eric J. Alm, Manimozhiyan Arumugam, Francesco Asnicar, Yang Bai, Jordan E. Bisanz, Kyle Bittinger, Asker Brejnrod, Colin J. Brislawn, C. Titus Brown, Benjamin J. Callahan, Andrés Mauricio Caraballo-Rodríguez, John Chase, Emily K. Cope, Ricardo Da Silva, Christian Diener, Pieter C. Dorrestein, Gavin M. Douglas, Daniel M. Durall, Claire Duvallet, Christian F. Edwardson, Madeleine Ernst, Mehrbod Estaki, Jennifer Fouquier, Julia M. Gauglitz, Sean M. Gibbons, Deanna L. Gibson, Antonio Gonzalez, Kestrel Gorlick, Jiarong Guo, Benjamin Hillmann, Susan Holmes, Hannes Holste, Curtis Huttenhower, Gavin A. Huttley, Stefan Janssen, Alan K. Jarmusch, Lingjing Jiang, Benjamin D. Kaehler, Kyo Bin Kang, Christopher R. Keefe, Paul Keim, Scott T. Kelley, Dan Knights, Irina Koester, Tomasz Kosciolek, Jorden Kreps, Morgan G. I. Langille, Joslynn Lee, Ruth Ley, Yong-Xin Liu, Erikka Loftfield, Catherine Lozupone, Massoud Maher, Clarisse Marotz, Bryan D. Martin, Daniel McDonald, Lauren J. McIver, Alexey V. Melnik, Jessica L. Metcalf, Sydney C. Morgan, Jamie T. Morton, Ahmad Turan Naimey, Jose A. Navas-Molina, Louis Felix Nothias, Stephanie B. Orchanian, Talima Pearson, Samuel L. Peoples, Daniel Petras, Mary Lai Preuss, Elmar Pruesse, Lasse Buur Rasmussen, Adam Rivers, Michael S. Robeson, Patrick Rosenthal, Nicola Segata, Michael Shaffer, Arron Shiffer, Rashmi Sinha, Se Jin Song, John R. Spear, Austin D. Swafford, Luke R. Thompson, Pedro J. Torres, Pauline Trinh, Anupriya Tripathi, Peter J. Turnbaugh, Sabah Ul-Hasan, Justin J. J. van der Hooft, Fernando Vargas, Yoshiki Vázquez-Baeza, Emily Vogtmann, Max von Hippel, William Walters, Yunhu Wan, Mingxun Wang, Jonathan Warren, Kyle C. Weber, Charles H. D. Williamson, Amy D. Willis, Zhenjiang Zech Xu, Jesse R. Zaneveld, Yilong Zhang, Qiyun Zhu, Rob Knight, and J. Gregory Caporaso. Reproducible, interactive, scalable and extensible microbiome data science using QIIME 2. *Nature Biotechnology*, 37(8):852–857, August 2019. ISSN 1546-1696. doi: 10.1038/s41587-019-0209-9.

[18] Benjamin J. Callahan, Paul J. McMurdie, Michael J. Rosen, Andrew W. Han, Amy Jo A. Johnson, and Susan P. Holmes. DADA2: High-resolution sample inference from Illumina amplicon data. *Nature Methods*, 13(7):581–583, July 2016. ISSN 1548-7105. doi: 10.1038/nmeth.3869.

[19] Christian Quast, Elmar Pruesse, Pelin Yilmaz, Jan Gerken, Timmy Schweer, Pablo Yarza, Jörg Peplies, and Frank Oliver Glöckner. The SILVA ribosomal RNA gene database project: Improved data processing and web-based tools. *Nucleic Acids Research*, 41(D1):D590–D596, January 2013. ISSN 0305-1048. doi: 10.1093/nar/gks1219.

[20] Pelin Yilmaz, Laura Wegener Parfrey, Pablo Yarza, Jan Gerken, Elmar Pruesse, Christian Quast, Timmy Schweer, Jörg Peplies, Wolfgang Ludwig, and Frank Oliver Glöckner. The SILVA and "All-species Living Tree Project (LTP)" taxonomic frameworks. *Nucleic Acids Research*, 42(D1):D643–D648, January 2014. ISSN 0305-1048. doi: 10.1093/nar/gkt1209.

[21] Nicole M. Davis, Diana M. Proctor, Susan P. Holmes, David A. Relman, and Benjamin J. Callahan. Simple statistical identification and removal of contaminant sequences in marker-gene and metagenomics data. *Microbiome*, 6(1):226, December 2018. ISSN 2049-2618. doi: 10.1186/s40168-018-0605-2.

[22] Paul J. McMurdie and Susan Holmes. Phyloseq: An R Package for Reproducible Interactive Analysis and Graphics of Microbiome Census Data. *PLOS ONE*, 8(4): e61217, April 2013. ISSN 1932-6203. doi: 10.1371/journal.pone.0061217.

[23] Jari Oksanen, F. Guillaume Blanchet, Michael Friendly, Roeland Kindt, Pierre Legendre, Dan McGlinn, Peter R. Minchin, R. B. O'Hara, Gavin L. Simpson, Peter Solymos, M. Henry H. Stevens, Eduard Szoecs, and Helene Wagner. Vegan: Community Ecology Package, 2020.

[24] Miquel De Cáceres and Pierre Legendre. Associations between species and groups of sites: Indices and statistical inference. *Ecology*, 90(12):3566–3574, 2009. ISSN 1939-9170. doi: 10.1890/08-1823.1.

[25] Stephen C Watts, Scott C Ritchie, Michael Inouye, and Kathryn E Holt. FastSpar: Rapid and scalable correlation estimation for compositional data. *Bioinformatics*, 35 (6):1064–1066, March 2019. ISSN 1367-4803. doi: 10.1093/bioinformatics/bty734.

[26] Gabor Csardi and Tamas Nepusz. The Igraph Software Package for Complex Network Research. *InterJournal*, Complex Systems:1695, November 2005.

[27] Paul Shannon, Andrew Markiel, Owen Ozier, Nitin S. Baliga, Jonathan T. Wang, Daniel Ramage, Nada Amin, Benno Schwikowski, and Trey Ideker. Cytoscape: A Software Environment for Integrated Models of Biomolecular Interaction Networks. *Genome Research*, 13(11):2498–2504, November 2003. ISSN 1088-9051, 1549-5469. doi: 10.1101/gr.1239303.

[28] Gary D. Bader and Christopher WV Hogue. An automated method for finding molecular complexes in large protein interaction networks. *BMC Bioinformatics*, 4(1): 2, January 2003. ISSN 1471-2105. doi: 10.1186/1471-2105-4-2.

[29] Masaru Konishi Nobu, Takashi Narihiro, Kyohei Kuroda, Ran Mei, and Wen-Tso Liu. Chasing the elusive Euryarchaeota class WSA2: Genomes reveal a uniquely fastidious methyl-reducing methanogen. *The ISME Journal*, 10(10):2478–2487, October 2016. ISSN 1751-7370. doi: 10.1038/ismej.2016.33.

[30] Daan R. Speth and Victoria J. Orphan. Metabolic marker gene mining provides insight in global mcrA diversity and, coupled with targeted genome reconstruction, sheds further light on metabolic potential of the Methanomassiliicoccales. *PeerJ*, 6: e5614, September 2018. ISSN 2167-8359. doi: 10.7717/peerj.5614.

[31] I-Min A Chen, Ken Chu, Krishnaveni Palaniappan, Anna Ratner, Jinghua Huang, Marcel Huntemann, Patrick Hajek, Stephan Ritter, Neha Varghese, Rekha Seshadri, Simon Roux, Tanja Woyke, Emiley A Eloe-Fadrosh, Natalia N Ivanova, and Nikos C Kyrpides. The IMG/M data management and analysis system v.6.0: New tools and advanced capabilities. *Nucleic Acids Research*, 49(D1):D751–D763, January 2021. ISSN 0305-1048. doi: 10.1093/nar/gkaa939.

[32] Stephen F. Altschul, Warren Gish, Webb Miller, Eugene W. Myers, and David J. Lipman. Basic local alignment search tool. *Journal of Molecular Biology*, 215(3):403–410, October 1990. ISSN 0022-2836. doi: 10.1016/S0022-2836(05)80360-2.

[33] Robert C. Edgar. Search and clustering orders of magnitude faster than BLAST. *Bioinformatics*, 26(19):2460–2461, October 2010. ISSN 1367-4803. doi: 10.1093/bioinformatics/btq461.

[34] Sean R. Eddy. Accelerated Profile HMM Searches. *PLOS Computational Biology*, 7(10): e1002195, October 2011. ISSN 1553-7358. doi: 10.1371/journal.pcbi.1002195.

[35] Benjamin Buchfink, Chao Xie, and Daniel H. Huson. Fast and sensitive protein alignment using DIAMOND. *Nature Methods*, 12(1):59–60, January 2015. ISSN 1548-7105. doi: 10.1038/nmeth.3176.

[36] Robert C. Edgar. MUSCLE: A multiple sequence alignment method with reduced time and space complexity. *BMC Bioinformatics*, 5(1):113, August 2004. ISSN 1471-2105. doi: 10.1186/1471-2105-5-113.

[37] Bui Quang Minh, Heiko A Schmidt, Olga Chernomor, Dominik Schrempf, Michael D Woodhams, Arndt von Haeseler, and Robert Lanfear. IQ-TREE 2: New Models and Efficient Methods for Phylogenetic Inference in the Genomic Era. *Molecular Biology and Evolution*, 37(5):1530–1534, May 2020. ISSN 0737-4038. doi: 10.1093/molbev/msaa015.

[38] Ivica Letunic and Peer Bork. Interactive Tree Of Life (iTOL) v5: An online tool for phylogenetic tree display and annotation. *Nucleic Acids Research*, 49(W1):W293–W296, July 2021. ISSN 0305-1048. doi: 10.1093/nar/gkab301.

[39] Andrey Prjibelski, Dmitry Antipov, Dmitry Meleshko, Alla Lapidus, and Anton Korobeynikov. Using SPAdes De Novo Assembler. *Current Protocols in Bioinformatics*, 70(1):e102, 2020. ISSN 1934-340X. doi: 10.1002/cpbi.102.

[40] Martin Steinegger, Milot Mirdita, and Johannes Söding. Protein-level assembly increases protein sequence recovery from metagenomic samples manyfold. *Nature Methods*, 16(7):603–606, July 2019. ISSN 1548-7105. doi: 10.1038/s41592-019-0437-4.

[41] Martin Steinegger and Johannes Söding. MMseqs2 enables sensitive protein sequence searching for the analysis of massive data sets. *Nature Biotechnology*, 35(11): 1026–1028, November 2017. ISSN 1546-1696. doi: 10.1038/nbt.3988.

[42] Sammy Pontrelli, Rachel Szabo, Shaul Pollak, Julia Schwartzman, Daniela Ledezma-Tejeida, Otto X. Cordero, and Uwe Sauer. Metabolic cross-feeding structures the assembly of polysaccharide degrading communities. *Science Advances*, 8(8):eabk3076, February 2022. doi: 10.1126/sciadv.abk3076.

[43] Ben J. Woodcroft, Samuel T. N. Aroney, Rossen Zhao, Mitchell Cunningham, Joshua A. M. Mitchell, Rizky Nurdiansyah, Linda Blackall, and Gene W. Tyson. Comprehensive taxonomic identification of microbial species in metagenomic data using SingleM and Sandpiper. *Nature Biotechnology*, 44(6):948–953, June 2026. ISSN 1546-1696. doi: 10.1038/s41587-025-02738-1.
